# ApoA-I mimetics reduce systemic and gut inflammation in chronic treated HIV

**DOI:** 10.1371/journal.ppat.1010160

**Published:** 2022-01-07

**Authors:** Maria Daskou, William Mu, Madhav Sharma, Hariclea Vasilopoulos, Rachel Heymans, Eleni Ritou, Valerie Rezek, Philip Hamid, Athanasios Kossyvakis, Shubhendu Sen Roy, Victor Grijalva, Arnab Chattopadhyay, Scott G. Kitchen, Alan M. Fogelman, Srinivasa T. Reddy, Theodoros Kelesidis

**Affiliations:** 1 Department of Medicine, Division of Infectious Diseases, David Geffen School of Medicine, University of California Los Angeles, Los Angeles, California, United States of America; 2 Department of Medicine, Division of Hematology and Oncology, David Geffen School of Medicine, University of California Los Angeles, Los Angeles, California, United States of America; 3 Department of Medicine, Division of Cardiology, David Geffen School of Medicine, University of California Los Angeles, Los Angeles, California, United States of America; 4 Department of Molecular and Medical Pharmacology, University of California Los Angeles, Los Angeles, California, United States of America; 5 Molecular Toxicology Interdepartmental Degree Program, University of California Los Angeles, Los Angeles, California, United States of America; Vaccine Research Center, UNITED STATES

## Abstract

Novel therapeutic strategies are needed to attenuate increased systemic and gut inflammation that contribute to morbidity and mortality in chronic HIV infection despite potent antiretroviral therapy (ART). The goal of this study is to use preclinical models of chronic treated HIV to determine whether the antioxidant and anti-inflammatory apoA-I mimetic peptides 6F and 4F attenuate systemic and gut inflammation in chronic HIV. We used two humanized murine models of HIV infection and gut explants from 10 uninfected and 10 HIV infected persons on potent ART, to determine the *in vivo* and *ex vivo* impact of apoA-I mimetics on systemic and intestinal inflammation in HIV. When compared to HIV infected humanized mice treated with ART alone, mice on oral apoA-I mimetic peptide 6F with ART had consistently reduced plasma and gut tissue cytokines (TNF-α, IL-6) and chemokines (CX3CL1) that are products of ADAM17 sheddase activity. Oral 6F attenuated gut protein levels of ADAM17 that were increased in HIV-1 infected mice on potent ART compared to uninfected mice. Adding oxidized lipoproteins and endotoxin (LPS) *ex vivo* to gut explants from HIV infected persons increased levels of ADAM17 in myeloid and intestinal cells, which increased TNF-α and CX3CL1. Both 4F and 6F attenuated these changes. Our preclinical data suggest that apoA-I mimetic peptides provide a novel therapeutic strategy that can target increased protein levels of ADAM17 and its sheddase activity that contribute to intestinal and systemic inflammation in treated HIV. The large repertoire of inflammatory mediators involved in ADAM17 sheddase activity places it as a pivotal orchestrator of several inflammatory pathways associated with morbidity in chronic treated HIV that make it an attractive therapeutic target.

## Author summary

Novel therapeutic strategies are needed to attenuate increased systemic and gut inflammation that contribute to morbidity and mortality in chronic HIV infection despite potent antiviral therapy. In this study we used preclinical models of chronic HIV (humanized mice and gut tissues from HIV infected persons) to determine whether antioxidant and anti-inflammatory agents called apoA-I mimetic peptides could attenuate systemic and gut inflammation in chronic HIV. Our preclinical data suggest that apoA-I mimetic peptides can target increased levels of a protein called ADAM17 and its proinflammatory activity that contributes to systemic and gut inflammation in HIV. The large repertoire of inflammatory mediators involved in ADAM17 sheddase activity places it as a pivotal orchestrator of several inflammatory pathways associated with morbidity in chronic treated HIV that make it an attractive therapeutic target.

## Introduction

Despite antiretroviral therapy (ART), chronic treated HIV is a state of systemic inflammation and immune activation [1]. Innate immunity biomarkers of inflammation including cytokines like interleukin-(IL)-1β[2], IL-6[3,4], tumor necrosis factor-α (TNF-α)[3,5], chemokines like C-X3-C Motif Chemokine Ligand 1 (CX3CL1)/Fractalkine [6,7] and biomarkers of monocyte/macrophage (M/M) activation such as soluble CD14 (sCD14) and sCD163[1,8,9] are predictors of morbidity in people with chronic treated HIV (PWH) [1–6,8,9]. M/M rather than T cell activation is considered a more clinically relevant predictor of morbidity in chronic treated HIV [1,8,9]. Thus, there is an enormous unmet need for novel therapeutic strategies to attenuate systemic inflammation and activation of innate immunity in chronic treated HIV.

Apolipoprotein A-I (apoA-I) mimetic peptides bind bioactive lipids and endotoxin (LPS) with higher affinity than apoA-I and may be novel therapeutic agents for treatment of inflammatory diseases including cardiovascular and inflammatory bowel disease and cancer [10–14]. We have shown that an apoA-I mimetic peptide called 4F improved *ex vivo* antioxidant/anti-inflammatory activities of HDL from HIV-1 infected individuals with suppressed viremia on potent ART [15]. 4F attenuates gut inflammation in murine models of gut inflammation when given orally [14]. Importantly, 4F has been tested in humans [16,17] and has a safety profile that would favor its clinical testing in HIV. Another peptide named 6F that is expressed as a transgene in tomatoes, when concentrated (Tg6F) and given orally, attenuates cancer, cardiovascular and inflammatory bowel disease in mice [11–14]. Tg6F acts in the intestine, is not absorbed intact in the blood and attenuates M/M activation and intestinal inflammation [11–14]. Preclinical studies need to validate the therapeutic use of apoA-I mimetic peptides in inflammation in HIV. Herein, we tested at the preclinical level whether the apoA-I mimetic peptides 6F and 4F may attenuate intestinal and systemic inflammation in chronic treated HIV.

To bypass limitations of preclinical animal models of chronic treated HIV, we recently described a robust translational preclinical approach, where we used both NOD.Cg-*Prkdc*^*scid*^*Il2rg*^*tm1Wjl*^/SzJ (NSG) and Graft versus host disease (GVHD)-resistant C57BL/6 recombination activating gene 2 (Rag2)γcCD47 triple knockout (TKO) BLT mice [18–23] on independent ART regimens and gut explants from HIV infected participants to demonstrate that apoA-I mimetics attenuate innate immune activation, gut barrier dysfunction, plasma and intestinal oxidized lipoproteins in chronic treated HIV [24]. Herein, using murine biospecimens (blood and gut tissues) and human biospecimens (gut tissues) from our recently published study [24], we demonstrate that apoA-I mimetics consistently reduce plasma and gut tissue cytokines (TNF-α, IL-6) and chemokines (CX3CL1) and gut protein levels of the chemokine receptor CX3CR1. ApoA-I mimetics also reduced gut protein levels of the A disintegrin and metalloprotease 17 (ADAM17), an inflammation-inducible enzyme that is responsible for the protease-driven shedding of TNF-α, CX3CL1 and sCD163 [25], one of the most robust biomarkers of innate immune activation that is strongly associated with mortality in chronic treated HIV [9]. We also show that apoA-I mimetics attenuate *ex vivo* lipopolysaccharide (LPS)- and oxidized lipoprotein-induced upregulation of the sheddase ADAM17 that mediates release of TNF-α and CX3CL1 in gut explants of uninfected and HIV infected participants on potent ART [25]. Collectively our pre-clinical data demonstrate the potential therapeutic use of apoA-I mimetics to target the cross-talk between oxidized lipoproteins, LPS and ADAM17 to attenuate proinflammatory intestinal and systemic responses in chronic treated HIV.

## Results

### Tg6F but not potent ART attenuated systemic inflammation in BLT models of chronic treated HIV

HIV infected BLT mice are an established preclinical model to study *in vivo* novel therapeutic agents for immune responses and inflammation in HIV [18–23]. We generated two independent (TKO and NSG) BLT models of humanized mice with functional human immune cells [24] that were mock-infected or infected with HIV-1 (HIV^+^) and then treated with clinically relevant ART (HIV^+^ART^+^) together with a concentrate of transgenic tomatoes expressing the apoA-I mimetic peptide 6F (HIV^+^ART^+^Tg6F^+^) or a concentrate of transgenic control tomatoes ((HIV^+^ART^+^EV^+^), as described in Materials and Methods. Using a sensitive Luminex immunoassay platform in plasma of BLT mice, we detected major human and murine cytokines [interleukin (IL)-1β, IL-6, IL-8, IL-10, tumor necrosis factor alpha (TNF-α)] and chemokines [C-C Motif Chemokine Ligand 2 (CCL2), CCL3, CCL5, C-X3-C Motif Chemokine Ligand 1 (CX3CL1), C-X-C Motif Chemokine Ligand 10 (CXCL10)] in plasma of both GVHD-prone NSG (S1A, S1B, S1C and S1D Fig) and GVHD-resistant TKO (S2A, S2B, S2C and S2D Fig) uninfected BLT mice and in the small intestine (SI) of GVHD-resistant uninfected TKO BLT mice (S2E and S2F Fig) treated with a concentrate of transgenic control tomatoes (HIV^-^EV^+^).

**Fig 1:**
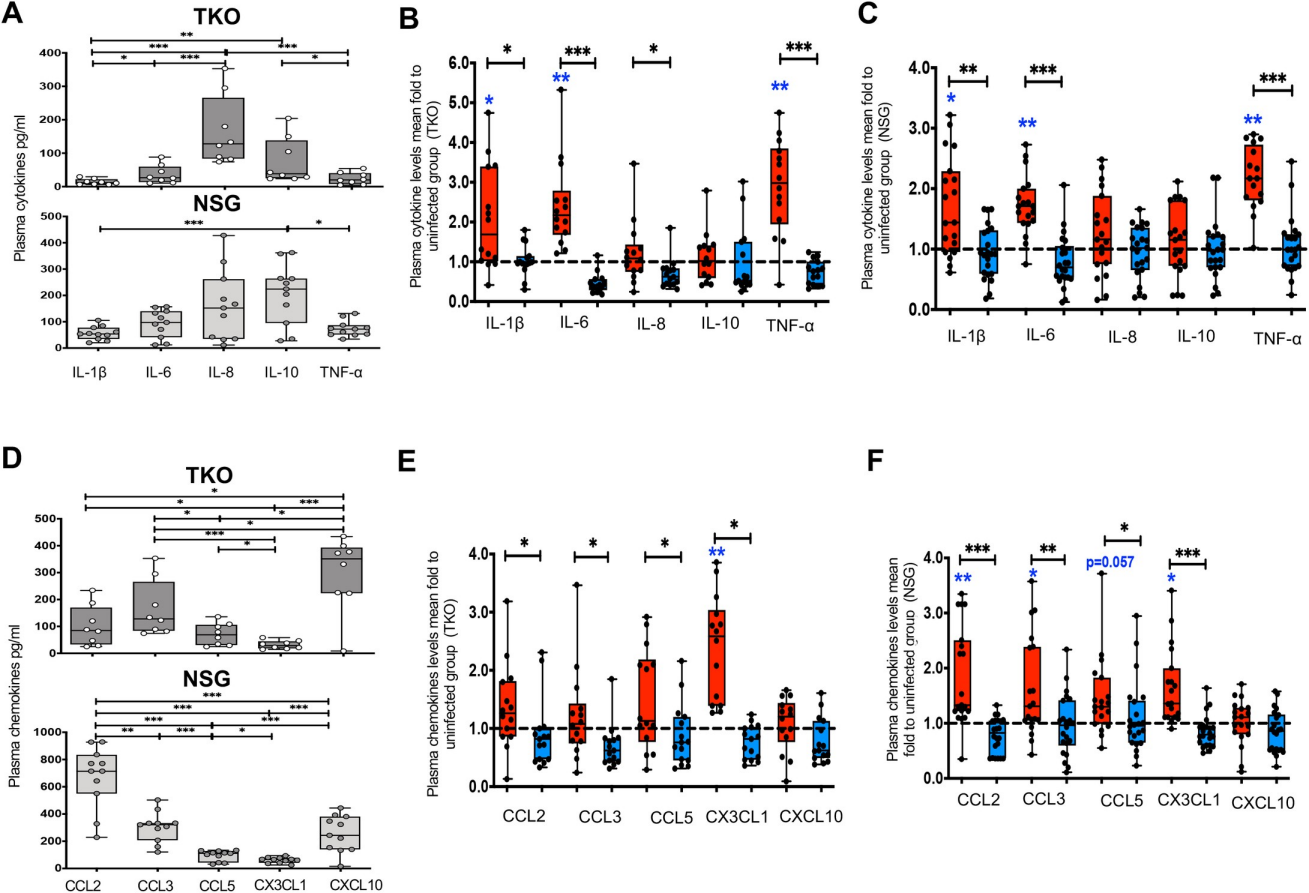
Tg6F attenuates increased systemic inflammation in humanized models of chronic treated HIV. NSG (n = 51) and TKO C57 (n = 37) humanized (BLT) mice were constructed, infected and treated with antiretroviral therapy (ART) and control transgenic tomato concentrate and/or oral ApoA-I mimetic peptide 6F in the form of transgenic tomato (Tg6F). Human cytokines [interleukin (IL)-1β, IL-6, IL-8, IL-10, tumor necrosis factor alpha (TNF-α)] and chemokines [C-C Motif Chemokine Ligand 2 (CCL2), CCL3, CCL5, C-X3-C Motif Chemokine Ligand 1 (CX3CL1), C-X-C Motif Chemokine Ligand 10 (CXCL10)] were determined in plasma by Luminex immunoassays after 16 weeks of HIV infection in the HIV^+^ART ^+^ group. The mean value of each measurement in HIV^+^ART^+^ (red color) and HIV^+^ART^+^/Tg6F^+^ (blue color) mice BLT mice was expressed as fold to the mean value of each measurement in uninfected BLT mice (within the same cohort). **A.** Fold changes of human cytokines in plasma of HIV^+^ART^+^ (n = 19) and HIV^+^ART^+^/Tg6F^+^ (n = 21) NSG mice compared to uninfected (n = 11) NSG BLT mice. **B.** Fold changes of human cytokines in plasma of HIV^+^ART^+^ (n = 14) and HIV^+^ART^+^/Tg6F^+^(n = 15) TKO BLT mice compared to uninfected (n = 8) TKO BLT mice. **C.** Fold changes of human chemokines in plasma of HIV^+^ART^+^ (n = 19) and HIV^+^ART^+^/Tg6F^+^ (n = 21) NSG BLT mice compared to uninfected (n = 11) NSG BLT mice. **D.** Fold changes of human chemokines in plasma of HIV^+^ART^+^ (n = 14) and HIV^+^ART^+^/Tg6F^+^(n = 15) TKO BLT mice compared to uninfected (n = 8) TKO BLT mice. Data represent box and whiskers with minimum, median and maximum values (*n* = 8–21 mice per group). Datapoints represent mean of at least 2 experimental replicates per mouse. The Kruskal Wallis was used to compared >2 groups and the Mann-Whitney test was used to compare 2 groups (*p < 0.05, **p < 0.01, ***p < 0.001). The asterisks in blue demonstrate the comparison relative to the uninfected group.

We then determined the impact of HIV-1 infection and ART on plasma cytokines and chemokines of innate immunity that contribute to systemic inflammation in chronic treated HIV [1,8,9]. Both NSG (Fig 1A) and TKO (Fig 1B) HIV^+^ ART^+^ BLT mice had increased plasma h-IL-1β, h-IL-6 and h-TNF-α compared to uninfected mice. Both NSG (S3A Fig) and TKO (S3B Fig) HIV^+^ ART^+^ BLT mice had increased plasma m-IL-1β and m-TNF-α compared to uninfected mice (S3A and S3B Fig). TKO HIV^+^ ART^+^ BLT mice had also increased plasma m-IL-6 compared to uninfected mice (S3B Fig). Both NSG (Fig 1C) and TKO (Fig 1D) HIV^+^ ART^+^ BLT mice had increased plasma h-CX3CL1 compared to uninfected mice. NSG HIV^+^ ART^+^ BLT mice had also increased plasma h-CCL2, h-CCL3 and tended to have increased h-CCL5 (p = 0.057) compared to uninfected mice (Fig 1C). Both NSG (S3C Fig) and TKO (S3C Fig) HIV^+^ ART^+^ BLT mice had increased plasma m-CX3CL1 compared to uninfected mice (S3C and S3D Fig). In both BLT models other human and murine plasma cytokines and chemokines did not increase in HIV^+^ ART^+^ mice compared to uninfected mice (Figs 1A, 1B, 1C, 1D and S3A, S3B, S3C, S3D). Overall, like our prior observations in studies of treated HIV that showed that potent ART does not attenuate inflammation [26], our data suggest that potent ART that suppressed plasma viremia [24] does not attenuate systemic inflammation in both BLT models of chronic treated HIV.

We hypothesized that oral apoA-I mimetic peptides like Tg6F that have anti-inflammatory properties [27] and attenuate proinflammatory microbial products like LPS and bioactive lipids [10,28,29], may attenuate intestinal and systemic inflammation in chronic treated HIV. To test our hypothesis, we started Tg6F treatment after the viremia was suppressed which was four weeks after the start of the experiment [24]. Tg6F was given in chow diet (0.06% by weight) to the HIV^+^ART^+^Tg6F^+^ TKO group. The uninfected (HIV^-^), HIV^+^ and HIV^+^ART^+^ mice were all fed with chow diet that contained control transgenic tomato concentrate (EV) to ensure that the effectiveness of Tg6F is due to the presence of 6F, which is not present in EV [11,13]. We also cotreated HIV^+^ART^+^ NSG BLT mice fed chow diet with 0.06% Tg6F (wt/wt) with systemic ART for 10 weeks. Both HIV^+^ART^+^ Tg6F^+^ TKO and NSG BLT mice treated with Tg6F therapy for up to 14 weeks had consistently reduced plasma human cytokines (IL-1β, IL-6, TNF-α) (Fig 1A and 1B) and human chemokines (CCL2, CCL3, CCL5, CX3CL1) (Fig 1C and 1D) compared to HIV^+^ART^+^ mice BLT mice. Both HIV^+^ART^+^ Tg6F^+^ TKO and NSG BLT mice treated with Tg6F therapy for up to 14 weeks had also consistently reduced plasma murine cytokines (IL-1β, IL-6, TNF-α) (S3A and S3B Fig) and murine chemokines (CX3CL1) (S3C and S3D Fig) compared to HIV^+^ART^+^ mice BLT mice. Tg6F therapy also tended to reduce (p = 0.075) and reduced murine CCL2 in NSG and TKO HIV^+^ART^+^ BLT mice, respectively (S3C and S3D Fig). Tg6F therapy did not impact human and murine IL-10 and CXCL10 in both NSG and TKO HIV^+^ART^+^ BLT mice (Figs 1A, 1B, 1C, 1D and S3A, S3B, S3C, S3D). Thus, Tg6F attenuated systemic inflammation in both BLT models of chronic treated HIV.

### Tg6F but not potent ART attenuated intestinal inflammation in BLT models of HIV

**Fig 2:**
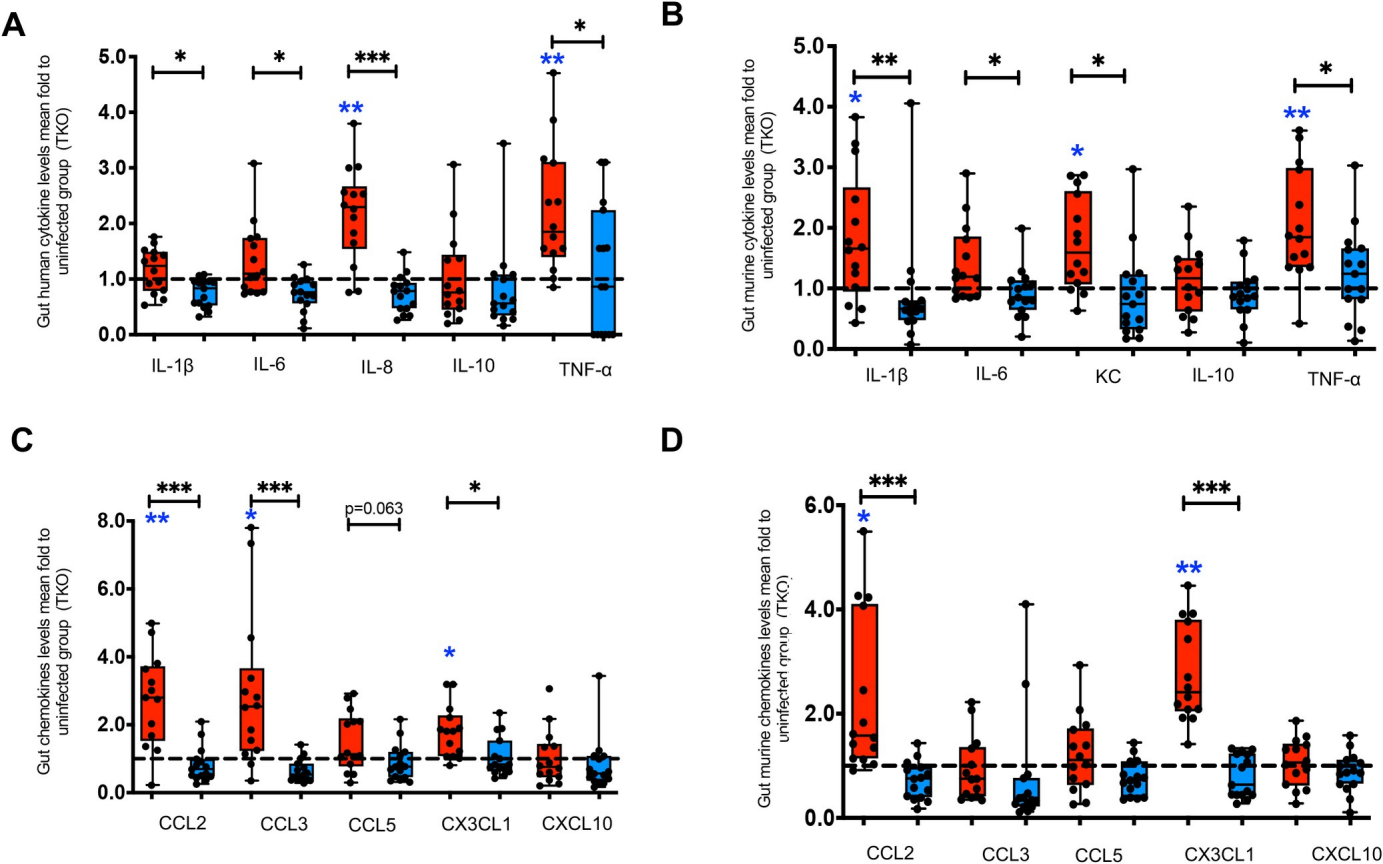
Tg6F attenuates gut inflammation in humanized mice with treated HIV infection. TKO C57 (n = 37) humanized (BLT) mice were constructed, infected and treated with antiretroviral therapy (ART) and control transgenic tomato concentrate and/or oral ApoA-I mimetic peptide 6F in the form of transgenic tomato (Tg6F). The small intestine from each mouse was collected and protein lysates were prepared from BLT mice after 16 weeks of HIV infection in the HIV^+^ART ^+^ group. Human and murine cytokines [interleukin (IL)-1β, IL-6, IL-8, IL-10, tumor necrosis factor alpha (TNF-α)] and chemokines [C-C Motif Chemokine Ligand 2 (CCL2), CCL3, CCL5, C-X3-C Motif Chemokine Ligand 1 (CX3CL1), C-X-C Motif Chemokine Ligand 10 (CXCL10)] were determined in gut tissue lysates by Luminex immunoassays after 16 weeks of HIV infection in the HIV^+^ART ^+^ group. The mean value of each measurement in HIV^+^ART^+^ (red color)(n = 14) and HIV^+^ART^+^/Tg6F^+^ (blue color)(n = 15) mice BLT mice was expressed as fold to the mean value of each measurement in uninfected (n = 8) BLT mice (within the same cohort). **A.** Fold changes of human cytokines in gut tissue lysates of HIV^+^ART^+^ and HIV^+^ART^+^/Tg6F^+^ TKO mice compared to uninfected BLT mice. **B.** Fold changes of murine cytokines in gut tissue lysates of HIV^+^ART^+^ and HIV^+^ART^+^/Tg6F^+^ TKO mice compared to uninfected BLT mice. **C.** Fold changes of human chemokines in gut tissue lysates of HIV^+^ART^+^ and HIV^+^ART^+^/Tg6F^+^ TKO mice compared to uninfected BLT mice. **D.** Fold changes of murine chemokines in gut tissue lysates of HIV^+^ART^+^ and HIV^+^ART^+^/Tg6F^+^ TKO mice compared to uninfected BLT mice. Data represent box and whiskers with minimum, median and maximum values (*n* = 8–15 mice per group). Datapoints represent mean of at least 2 experimental replicates per mouse. The Kruskal Wallis was used to compared >2 groups and the Mann-Whitney test was used to compare 2 groups (*p < 0.05, **p < 0.01, ***p < 0.001). The asterisks in blue demonstrate the comparison relative to the uninfected group.

Gut inflammation contributes to gut barrier dysfunction and systemic inflammation in chronic treated HIV [1,8,9,30]. Given that apoA-I mimetics act in the small intestine (SI) and the gut to attenuate systemic inflammation in murine models of inflammatory diseases [11–14], we further assessed gut inflammation in BLT models of chronic treated HIV. To minimize confounding effect of GVHD on gut inflammation in NSG BLT mice and given limited amount of total human protein in intestinal humanized mouse tissue, we used sensitive multiplex Luminex immunoassays to assess several major human cytokines and chemokines at the SI of GVHD-resistant TKO BLT mice (S2E and S2F Fig). HIV^+^ART^+^ TKO BLT mice had increased cytokines (h-IL-8, KC, h-TNF-α and m-TNF-α) (Fig 2A and 2B) and chemokines (h-CCL2, m-CCL2, h-CCL3, h- CX3CL1, m-CX3CL1) (Fig 2C and 2D) compared to uninfected TKO BLT mice. Overall, in both blood and intestine, potent ART did not attenuate increased intestinal levels of human and murine CX3CL1 and TNF-α in HIV-infected ART-treated BLT mice compared to uninfected mice. In the HIV^+^ART^+^ group, protein levels of m-TNF-α and m-CX3CL1 in gut, that are known instigators of gut barrier dysfunction [31], were associated with total protein levels of plasma sCD14 and m-IFABP (S4A, S4B, S4C, S4D, S4E, S4F, S4G and S4H Fig). Thus, increased TNF-α and CX3CL1 in gut and blood were associated with gut barrier dysfunction and inflammation in both BLT models of chronic treated HIV despite potent ART.

CX3CL1 was the most abundant human *and* murine chemokine that was consistently elevated in BLT mouse models of chronic treated HIV and is known to contribute to inflammation [32], gut barrier function [33], HIV progression and end organ disease [6,34,35]. The major chemokine receptor of CX3CL1 in M/M is CX3CR1 and is known to interact with bioactive lipids [36] and contribute to gut inflammation [37]. To further confirm our data that increased CX3CL1 levels in the gut contributes to gut inflammation in HIV infected BLT mice, we used a density gradient- and collagenase-free method to isolate the relatively less abundant human myeloid cells and flow cytometry to determine protein levels of CX3CR1 in single cell suspension from SI of TKO BLT mice exposed to HIV and ART. Gating strategy for assessment of intestinal cells in TKO BLT mice is shown in S5 Fig. Compared to uninfected mice, HIV^+^ART^+^ TKO had increased membrane protein expression (MFI) of hCX3CR1 in hCD33^+^ gut tissue myeloid cells and tended to have increased murine m-CX3CR1 in mCD11b^+^ gut tissue myeloid cells (S6A, S6B and S6C Fig). Thus, consistent with prior data that increased CX3CL1-CX3CR1 axis contributes to gut inflammation [37], the CX3CL1-CX3CR1 axis was upregulated in myeloid intestinal cells in HIV infected BLT mice.

Given that Tg6F is not systemically absorbed and works in the gut [11,13,14], we hypothesized that Tg6F attenuates both intestinal and systemic inflammation in BLT models of chronic treated HIV. HIV^+^ART^+^ Tg6F^+^ TKO BLT mice had reduced intestinal human and murine cytokines (IL-1β, IL-6, IL-8, KC, TNF-α) (Fig 2A and 2B), chemokines (CCL2, CX3CL1)(Fig 2C and 2D) and h-CCL3 (Fig 2C) compared to EV-fed HIV^+^ART^+^ BLT mice. In the HIV^+^ART^+^Tg6F^+^ group, total protein levels of m-TNF-α and m-CX3CL1 in SI were not associated with plasma sCD14 and m-IFABP (S4 Fig). Compared to EV-fed HIV^+^ART^+^ TKO BLT mice, the Tg6F-fed HIV^+^ART^+^ TKO BLT mice expressed lower levels of CX3CR1 in human and murine myeloid intestinal cells (S6B and S6C Fig) in SI. Thus, confirming our previous reports that Tg6F attenuates intestinal inflammation [11,13,14], Tg6F attenuated several proinflammatory cytokines and chemokines at both the gut and blood level and intestinal CX3CR1 in BLT models of chronic treated HIV.

### Tg6F reduced increased protein levels of ADAM17 in the intestine of BLT mice with chronic HIV

**Fig 3:**
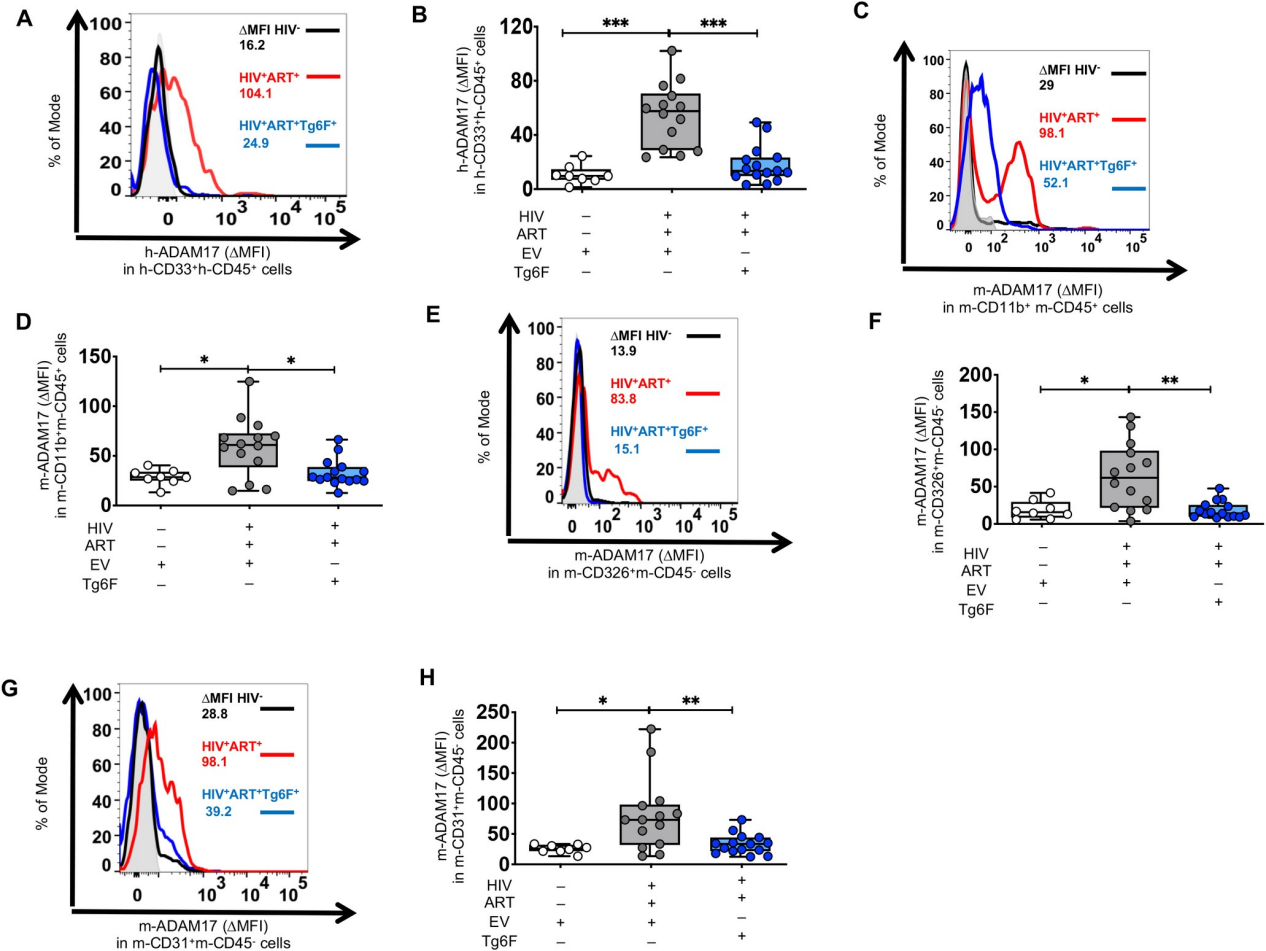
Tg6F attenuates increases in protein levels of ADAM17 in intestinal cells of humanized mice with treated HIV infection. TKO C57 (n = 45) humanized mice were constructed, infected with HIV and treated with antiretroviral therapy (ART) and control transgenic tomato concentrate and/or oral ApoA-I mimetic peptide 6F in the form of transgenic tomato (Tg6F). Single cell suspension from gut was prepared and cellular protein levels of ADAM17 were determined by flow cytometry in intestinal cells. **A.** Representative data of change in membrane protein expression of human ADAM17 in human h-CD33^+^h-CD45^+^ viable single myeloid immune cells are shown. ΔMFI represents a shift in median fluorescence intensity of a positive cell population compared to a negative cell population within each sample. The negative cell population was defined based on a fluorescence minus one negative control for staining as shown in light filled grey histogram). **B.** Summary of data for **(A). C.** Representative data of ΔMFI of murine ADAM17 in murine CD11b^+^CD45^+^ myeloid immune cells are shown**. D.** Summary of data for (**C**). **E.** Representative data of ΔMFI of murine ADAM17 in murine CD326^+^CD45^-^ epithelial cells are shown**. F.** Summary of data for (**E**). **G.** Representative data of ΔMFI of murine ADAM17 in murine CD31^+^CD45^-^ endothelial cells are shown**. H.** Summary of data for (**G**). Data represent box and whiskers with minimum, median and maximum values (*n* = 8–22 mice per group). Datapoints represent mean of at least 2 experimental replicates per mouse. The Kruskal Wallis was used to compared >2 groups and the Mann-Whitney test was used to compare 2 groups (*p < 0.05, **p < 0.01, ***p < 0.001).

We have shown both NSG and TKO HIV^+^ART^+^ BLT mice had increased biomarkers of human M/M activation (h-sCD14, h-sCD163) and gut barrier dysfunction (IFABP) compared to uninfected mice [24]. Notably, most of the measures of systemic inflammation that were *consistently* elevated in *both* BLT models of chronic treated HIV at the blood and gut level, are products of the sheddase activity of the protease ADAM17. ADAM17 is present in macrophages and enterocytes (to impact I-FABP)[25,38–40] and mediates the release of sCD14, sCD163, TNF-α, IL-6, CX3CL1, IFABP by M/M and/or enterocytes [41–45]. We therefore hypothesized that the ADAM17 protein is upregulated in gut tissue of HIV^+^ART^+^ mice. To test our hypothesis, we used flow cytometry to determine protein levels of ADAM17 in single cell suspension from SI of GVHD resistant TKO BLT mice exposed to HIV and ART. Protein levels of ADAM17 were increased in human h-CD33^+^h-CD45^+^ myeloid cells (Fig 3A and 3B), murine CD11b^+^CD45^+^ myeloid immune cells (Fig 3C and 3D), murine CD326^+^CD45^-^ epithelial cells (Fig 3E and 3F) and murine CD31^+^CD45^-^ endothelial cells (Fig 3G and 3H) of HIV^+^ART^+^ TKO BLT mice compared to uninfected mice. Thus, ADAM17 is a key proinflammatory protein that was consistently increased in myeloid, epithelial and endothelial cells in the intestine of BLT mice with chronic treated HIV.

Given that apoA-I mimetics bind LPS and reduce formation of oxidized lipoproteins that are inducers of ADAM17[25,46], we then investigated whether Tg6F reduces ADAM17 levels in BLT mouse models of chronic treated HIV. Tg6F decreased cellular protein levels of ADAM17 in human and murine myeloid cells and murine epithelial and endothelial cells of HIV^+^ART^+^Tg6F^+^ BLT mice to levels similar to cellular levels of HIV^-^ BLT mice (Fig 3A, 3B, 3C, 3D, 3E, 3F, 3G, 3H). Overall, our data demonstrated that Tg6F attenuated protein levels of ADAM17 in the gut that contributes to increased gut and systemic inflammation, in chronic treated HIV.

### 4F and 6F attenuate *ex vivo* ADAM17 levels and associated proinflammatory sheddase activity in gut explants of HIV infected ART-treated participants

**Fig 4:**
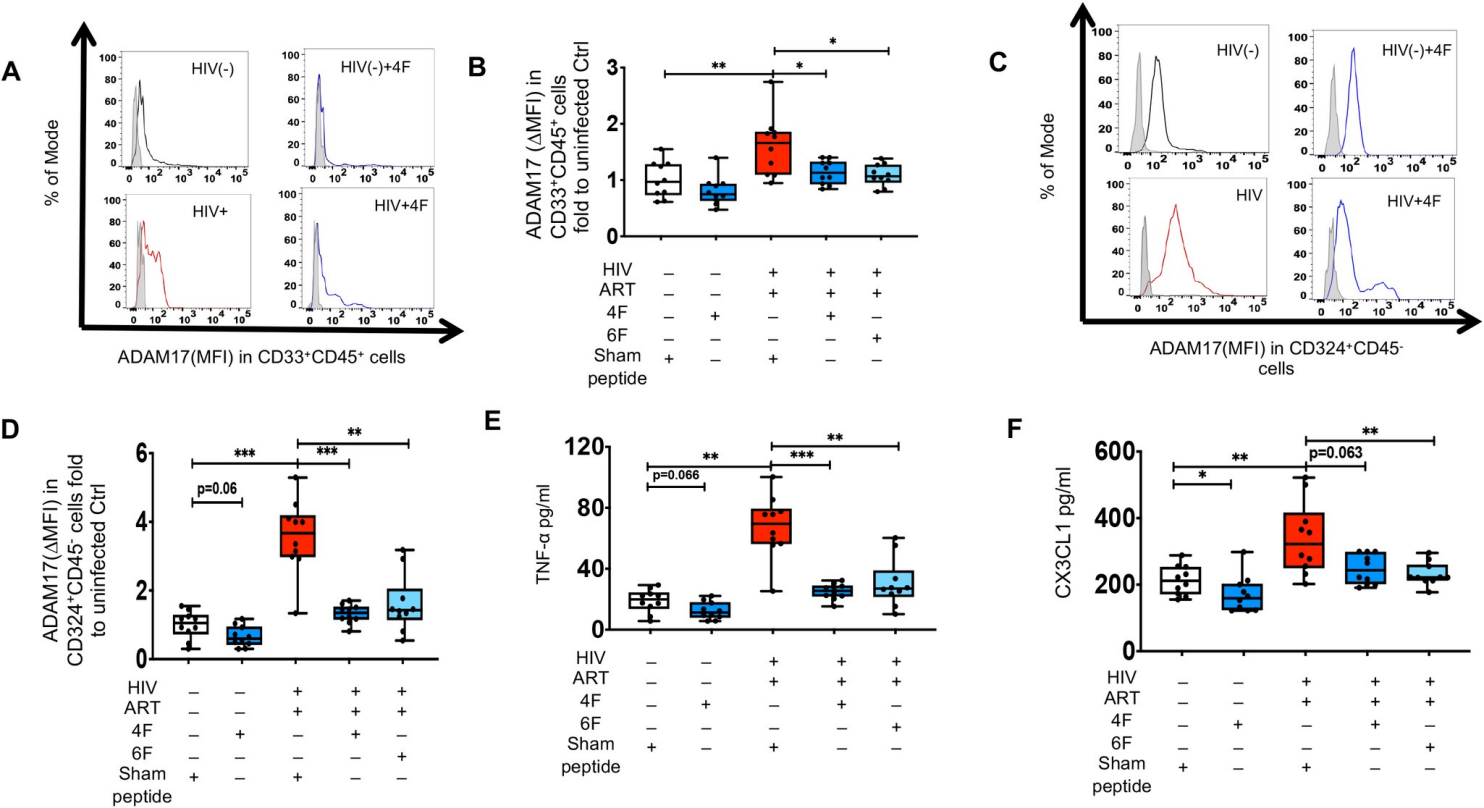
ApoA-I mimetic peptides attenuate *ex vivo* proteins levels of ADAM17 and associated cytokines and chemokines in gut explants of uninfected and HIV infected participants. Gut biopsies were obtained from uninfected (n = 10) and HIV infected participants on potent antiretroviral therapy (ART) (n = 10) and gut explants were treated with 4F or 6F apoA-I mimetic peptides or sham peptide for 72 hours as in Materials and Methods. Gut biopsies from the same participants were used for experiments with treatments. **A-D.** Protein levels of A disintegrin and metalloprotease 17 (ADAM17) was detected in myeloid CD33^+^CD45^+^ **(A, B**) and CD324^+^CD45^-^ epithelial cells **(C, D**) isolated from gut biopsies obtained from uninfected and HIV infected participants treated as shown. **A, C**. Representative data of flow cytometry data of protein levels (Median fluorescence intensity) of ADAM17 are shown for myeloid CD33^+^CD45^+^ **(A**) and CD324^+^CD45^-^ epithelial cells **(C**). ΔMFI represents a shift in median fluorescence intensity of a positive cell population compared to a negative cell population within each sample. The negative cell population was defined based on a fluorescence minus one negative control for staining as shown in light filled grey histogram. **B, D.** Summary data for protein levels of ADAM17 in intestinal myeloid CD33^+^CD45^+^ (**B**) and CD324^+^CD45^-^ epithelial cells (**D**) from gut biopsies treated as shown. **E, F.** Supernatants were collected at 72 hours and Luminex immunoassays were used to determine protein levels of cytokines [tumor necrosis factor alpha (TNF-α)](**E)** and chemokines [C-X3-C Motif Chemokine Ligand 1 (CX3CL1)] (**F)**, that are mediated by ADAM17 sheddase activity. Data represent box and whiskers with minimum, median and maximum values. Datapoints represent mean of 3 gut biopsies per participant. The Mann-Whitney test was used to compare 2 groups (*p < 0.05, **p < 0.01, ***p < 0.001).

Both TNF-α and CX3CL1 are major products of ADAM17 sheddase [25] that were consistently elevated in blood and gut of HIV^+^ART^+^ BLT mice and are known to contribute to inflammation [32] and gut barrier function [33]. Given known limitations of BLT mouse models of chronic treated HIV [24] and that Tg6F is not yet available for clinical trial in humans, we used colon tissues of uninfected (n = 10) and HIV infected (n = 10) ART-treated 50–60 years old white men without clinical morbidity to further validate our results that apoA-I mimetic peptides attenuate increased ADAM17 protein levels and its sheddase activity (protein levels and TNF-α and CX3CL1) in chronic treated HIV. Gut explants of HIV^+^ participants had higher protein levels of ADAM17 in myeloid (Fig 4A and 4B) and epithelial cells (Fig 4C and 4D) and higher protein levels of secreted TNF-α (Fig 4E) and CX3CL1 (Fig 4F) compared to uninfected participants. 4F tended (0.05<p<0.10) to reduce *ex vivo* ADAM17 protein levels in intestinal epithelial cells (Fig 4C and 4D) and secretion of TNF-α (Fig 4E) in supernatants of gut explants from uninfected participants. 4F reduced *ex vivo* secretion of CX3CL1 (Fig 4F) in supernatants of gut explants from uninfected participants. Unlike treatment with the sham peptide, and similar to our prior data with sCD14 and sCD163 [24], treatment of gut explants with 4F or 6F for 72 hours, attenuated *ex vivo* increased levels of ADAM17 in myeloid (Fig 4A and 4B) and epithelial cells (Fig 4C and 4D) isolated from gut explants of HIV^+^ participants. 4F and 6F also attenuated secreted TNF-α (Fig 4E) and CX3CL1 (Fig 4F) in supernatants of gut explants of HIV^+^ participants. Overall, our complementary BLT mouse and *ex vivo* human studies, demonstrated that apoA-I mimetic peptides attenuate ADAM17 protein levels and its sheddase products, CX3CL1 and TNF-α that promote intestinal and systemic inflammation in chronic treated HIV.

### 4F and 6F attenuate proinflammatory effect of LPS on intestinal ADAM17 levels and associated sheddase activity in chronic treated HIV

**Fig 5:**
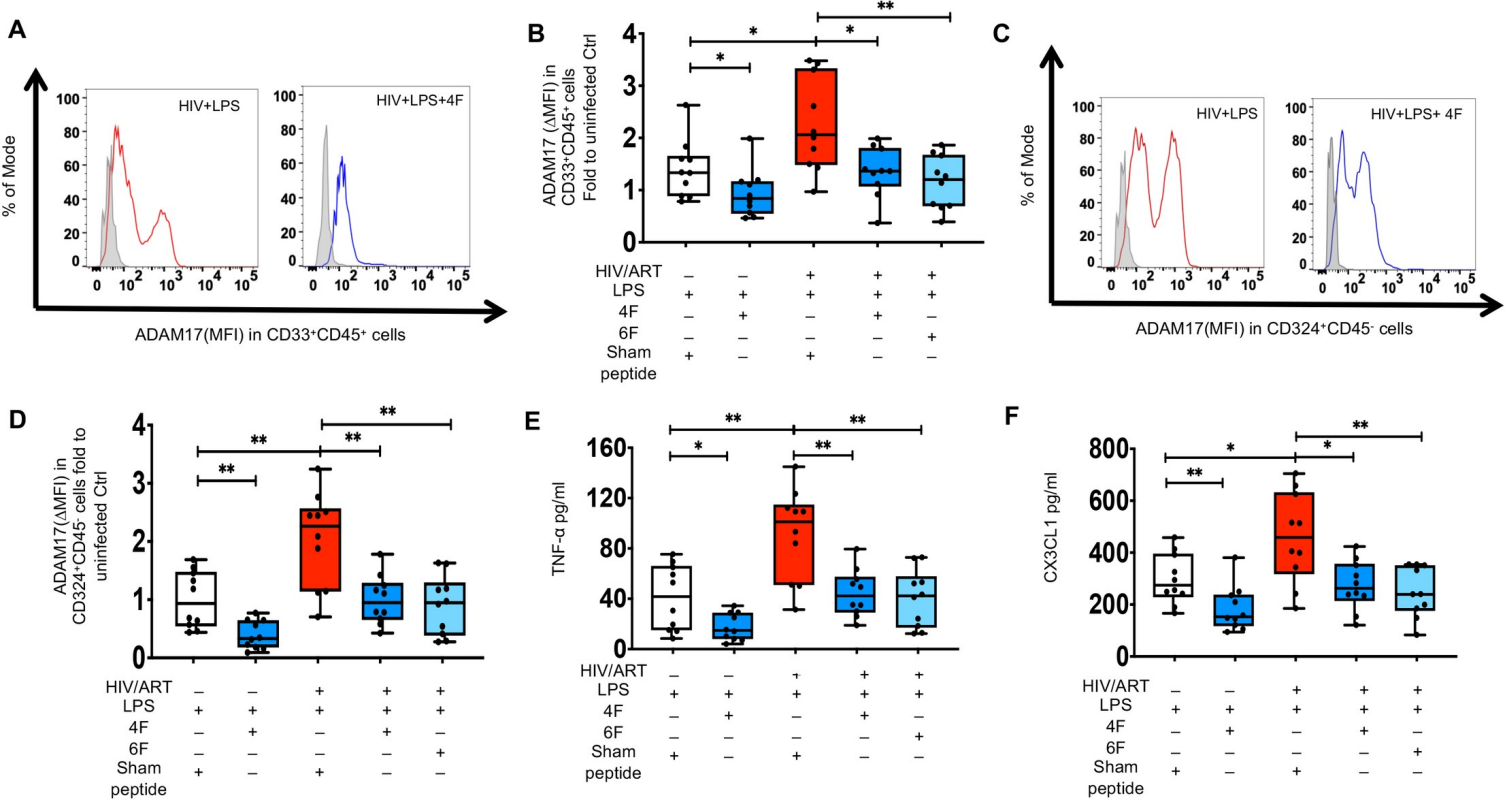
*Ex vivo* impact of ApoA-I mimetic peptides and endotoxin (LPS) on proteins levels of ADAM17 and associated cytokines and chemokines in gut explants of uninfected and HIV infected participants. Gut biopsies were obtained from uninfected (n = 10) and HIV infected participants on potent ART (n = 10) and gut explants were treated with endotoxin (LPS) for 48 hours and 4F or 6F apoA-I mimetic peptides or sham peptide for 72 hours as in Materials and Methods. Gut biopsies from the same participants were used for experiments with treatments. **A-D.** Protein levels of A disintegrin and metalloprotease 17 (ADAM17) was detected in myeloid CD33^+^CD45^+^ **(A, B**) and CD324^+^CD45^-^ epithelial cells **(C, D**) isolated from gut biopsies obtained from uninfected and HIV infected participants treated as shown. **A, C**. Representative data of flow cytometry data of protein levels (Median fluorescence intensity) of ADAM17 are shown for myeloid CD33^+^CD45^+^. **(A**) and CD324^+^CD45^-^ epithelial cells **(C**). ΔMFI represents a shift in median fluorescence intensity of a positive cell population compared to a negative cell population within each sample. The negative cell population was defined based on a fluorescence minus one negative control for staining as shown in light filled grey histogram. **B, D.** Summary data for protein levels of ADAM17 in intestinal myeloid CD33^+^CD45^+^ (**B**) and CD324^+^CD45^-^ epithelial cells (**D**) from gut biopsies treated as shown. **E, F.** Supernatants were collected at 72 hours and Luminex immunoassays were used to determine protein levels of cytokines [tumor necrosis factor alpha (TNF-α)](**E)** and chemokines [C-X3-C Motif Chemokine Ligand 1 (CX3CL1)] (**F)**, that are mediated by ADAM17 sheddase activity. Data represent box and whiskers with minimum, median and maximum values. Datapoints represent mean of 3 gut biopsies per participant. The Mann-Whitney test was used to compare 2 groups (*p < 0.05, **p < 0.01, ***p < 0.001).

We have previously shown that Tg6F attenuates plasma LPS in BLT mouse models of chronic treated HIV [24]. Given that apoA-I mimetics bind LPS, an inducer of ADAM17[25,47–49], we hypothesized that the inhibitory effect of apoA-I mimetic peptides on ADAM17 is mediated through LPS. Consistent with this hypothesis, increased protein levels of murine ADAM17 in murine epithelial cells of HIV infected BLT mice on potent ART were positively associated with increased protein levels of biomarkers of gut barrier dysfunction (sCD14, I-FABP) and immune activation (sCD14, sCD163) (S1 Table). Protein cellular levels of ADAM17 did not correlate with plasma h-sCD14, h-sCD163, m-IFABP in HIV^+^ART^+^Tg6F^+^ TKO BLT mice with an intervention that is known to bind LPS [14](S1 Table). We then tested whether LPS triggers *ex vivo* protein levels of ADAM17 and secretion of TNF-α and CX3CL1 from colon tissues of uninfected and HIV infected ART-treated persons. Gut explants of HIV^+^ participants treated with LPS had higher protein levels of ADAM17 in myeloid CD33^+^CD45^+ (^Fig 5A and 5B) and CD324^+^CD45^-^ epithelial cells (Fig 5C and 5D) compared to vehicle controls (Fig 4A, 4B, 4C and 4D). Gut explants of HIV^+^ participants treated with LPS had also higher protein levels of TNF-α (Fig 5E) and CX3CL1 (Fig 5F) compared to vehicle controls (Fig 4E and 4F). These results were not observed in LPS-treated gut explants from uninfected persons (Figs 4B, 4D, 4E, 4F and 5B, 5D, 5E, 5F). Both 4F and 6F reduced ADAM17 in myeloid cells (Fig 5A and 5B) and epithelial cells (Fig 5C and 5D) and secretion of TNF-α (Fig 5E) and CX3CL1 (Fig 5F) from LPS-treated gut explants from both uninfected and HIV^+^ participants. Overall, our data suggest that apoA-I mimetic peptides attenuate the crosstalk between increased gut barrier dysfunction, LPS, ADAM17 and associated production of TNF-α and CX3CL1 in gut tissue that contribute to gut and systemic inflammation in chronic treated HIV.

### 4F and 6F attenuate proinflammatory effect of oxidized lipoproteins on intestinal ADAM17 levels and associated sheddase activity in chronic treated HIV

**Fig 6:**
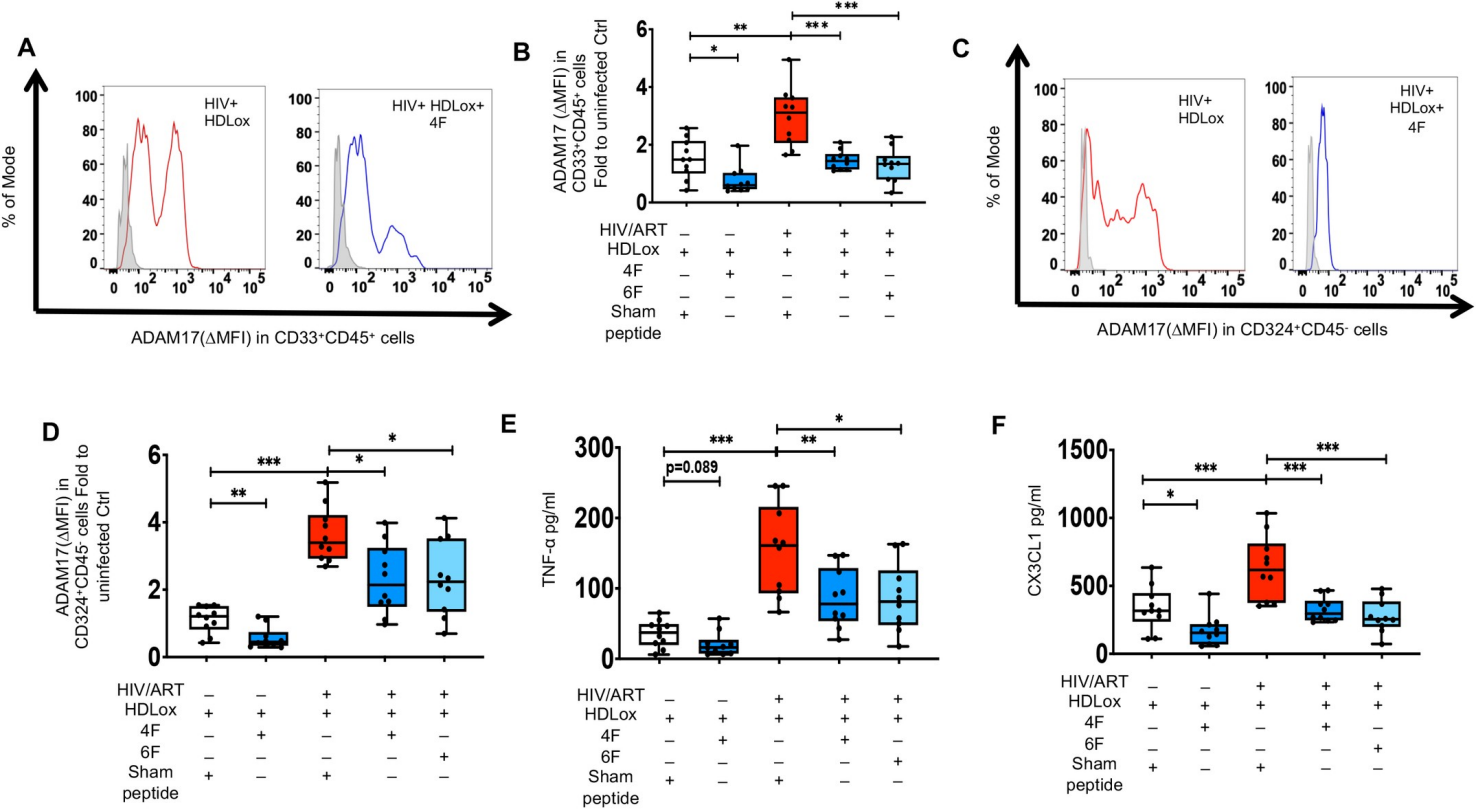
*Ex vivo* impact of ApoA-I mimetic peptides and oxidized high density lipoprotein (HDLox) on proteins levels of ADAM17 and associated cytokines and chemokines in gut explants of uninfected and HIV infected participants. Gut biopsies were obtained from uninfected (n = 10) and HIV infected participants on potent antiretroviral therapy (ART) (n = 10) and gut explants were treated with HDLox for 48 hours and 4F or 6F apoA-I mimetic peptides or sham peptide for 72 hours as in Materials and Methods. Gut biopsies from the same participants were used for experiments with treatments. **A-D.** Protein levels of A disintegrin and metalloprotease 17 (ADAM17) was detected in myeloid CD33^+^CD45^+^ **(A, B**) and CD324^+^CD45^-^ epithelial cells **(C, D**) isolated from gut biopsies obtained from uninfected and HIV infected participants treated as shown. **A, C**. Representative data of flow cytometry data of protein levels (Median fluorescence intensity) of ADAM17 are shown for myeloid CD33^+^CD45^+^**. (A**) and CD324^+^CD45^-^ epithelial cells **(C**). ΔMFI represents a shift in median fluorescence intensity of a positive cell population compared to a negative cell population within each sample. The negative cell population was defined based on a fluorescence minus one negative control for staining as shown in light filled grey histogram. **B, D.** Summary data for protein levels of ADAM17 in intestinal myeloid CD33^+^CD45^+^ (**B**) and CD324^+^CD45^-^ epithelial cells (**D**) from gut biopsies treated as shown. **E, F.** Supernatants were collected at 72 hours and Luminex immunoassays were used to determine protein levels of cytokines [tumor necrosis factor alpha (TNF-α)](**E)** and chemokines [C-X3-C Motif Chemokine Ligand 1 (CX3CL1)] (**F)**, that are mediated by ADAM17 sheddase activity. Data represent box and whiskers with minimum, median and maximum values. Datapoints represent mean of 3 gut biopsies per participant. The Mann-Whitney test was used to compare 2 groups (*p < 0.05, **p < 0.01, ***p < 0.001).

**Fig 7:**
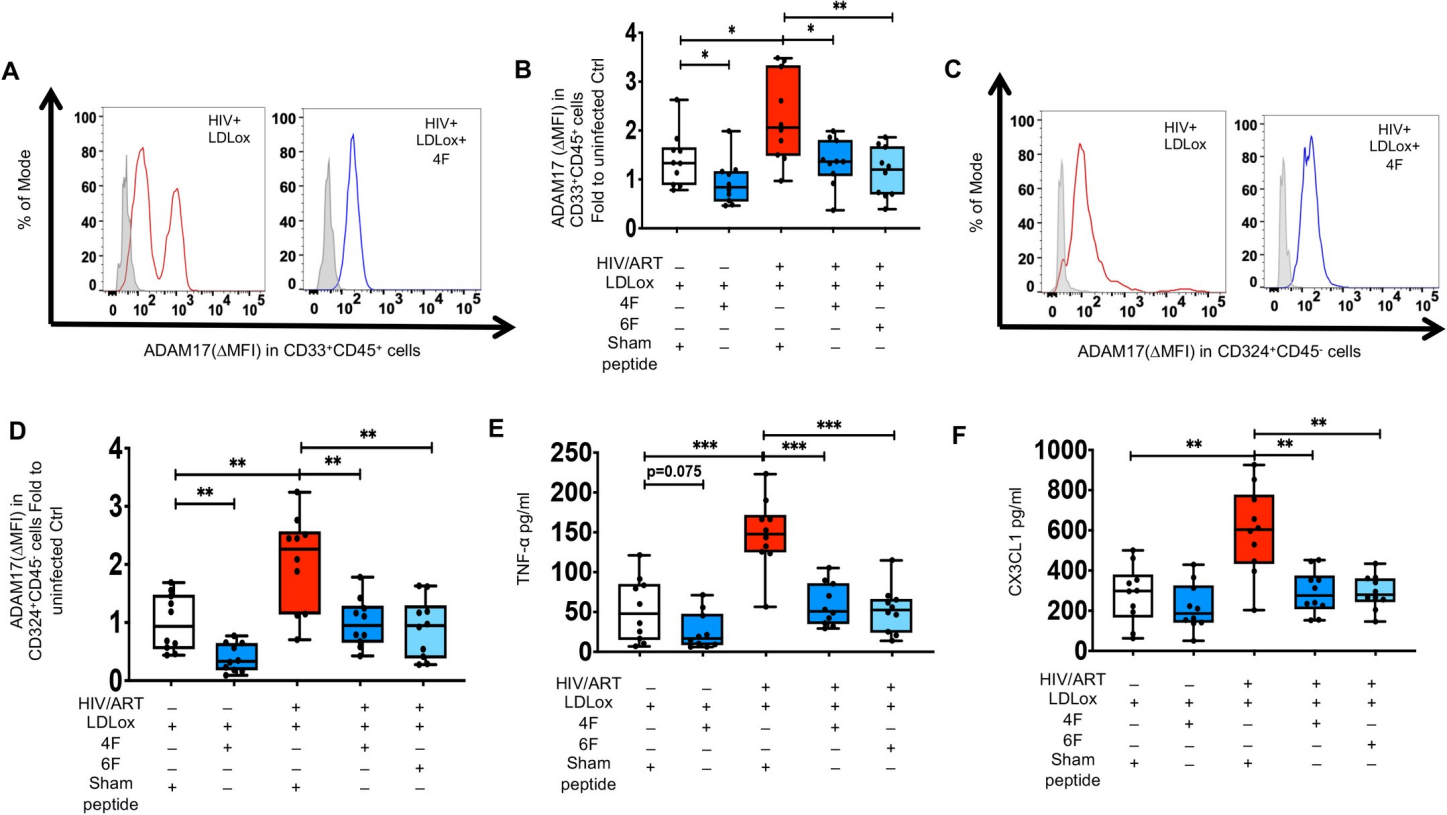
*Ex vivo* impact of ApoA-I mimetic peptides and oxidized low density lipoprotein (LDLox) on proteins levels of ADAM17 and associated cytokines and chemokines in gut explants of uninfected and HIV infected participants. Gut biopsies were obtained from uninfected (n = 10) and HIV infected participants on potent antiretroviral therapy (ART)(n = 10) and gut explants were treated with HDLox for 48 hours and 4F or 6F apoA-I mimetic peptides or sham peptide for 72 hours as in Materials and Methods. Gut biopsies from the same participants were used for experiments with treatments. **A-D.** Protein levels of A disintegrin and metalloprotease 17 (ADAM17) was detected in myeloid CD33^+^CD45^+^ **(A, B**) and CD324^+^CD45^-^ epithelial cells **(C, D**) isolated from gut biopsies obtained from uninfected and HIV infected participants treated as shown. **A, C**. Representative data of flow cytometry data of protein levels (Median fluorescence intensity) of ADAM17 against a negative stain control (fluorescence minus one control shown in light grey) are shown for myeloid CD33^+^CD45^+^ **(A**) and CD324^+^CD45^-^ epithelial cells **(C**). **B, D.** Summary data for protein levels of ADAM17 in intestinal myeloid CD33^+^CD45^+^ (**B**) and CD324^+^CD45^-^ epithelial cells (**D**) from gut biopsies treated as shown. **E, F.** Supernatants were collected at 72 hours and Luminex immunoassays were used to determine protein levels of cytokines [tumor necrosis factor alpha (TNF-α)](**E)** and chemokines [C-X3-C Motif Chemokine Ligand 1 (CX3CL1)] (**F)**, that are mediated by ADAM17 sheddase activity. Data represent box and whiskers with minimum, median and maximum values. Datapoints represent mean of 3 gut biopsies per participant. The Mann-Whitney test was used to compare 2 groups (*p < 0.05, **p < 0.01, ***p < 0.001).

**Fig 8:**
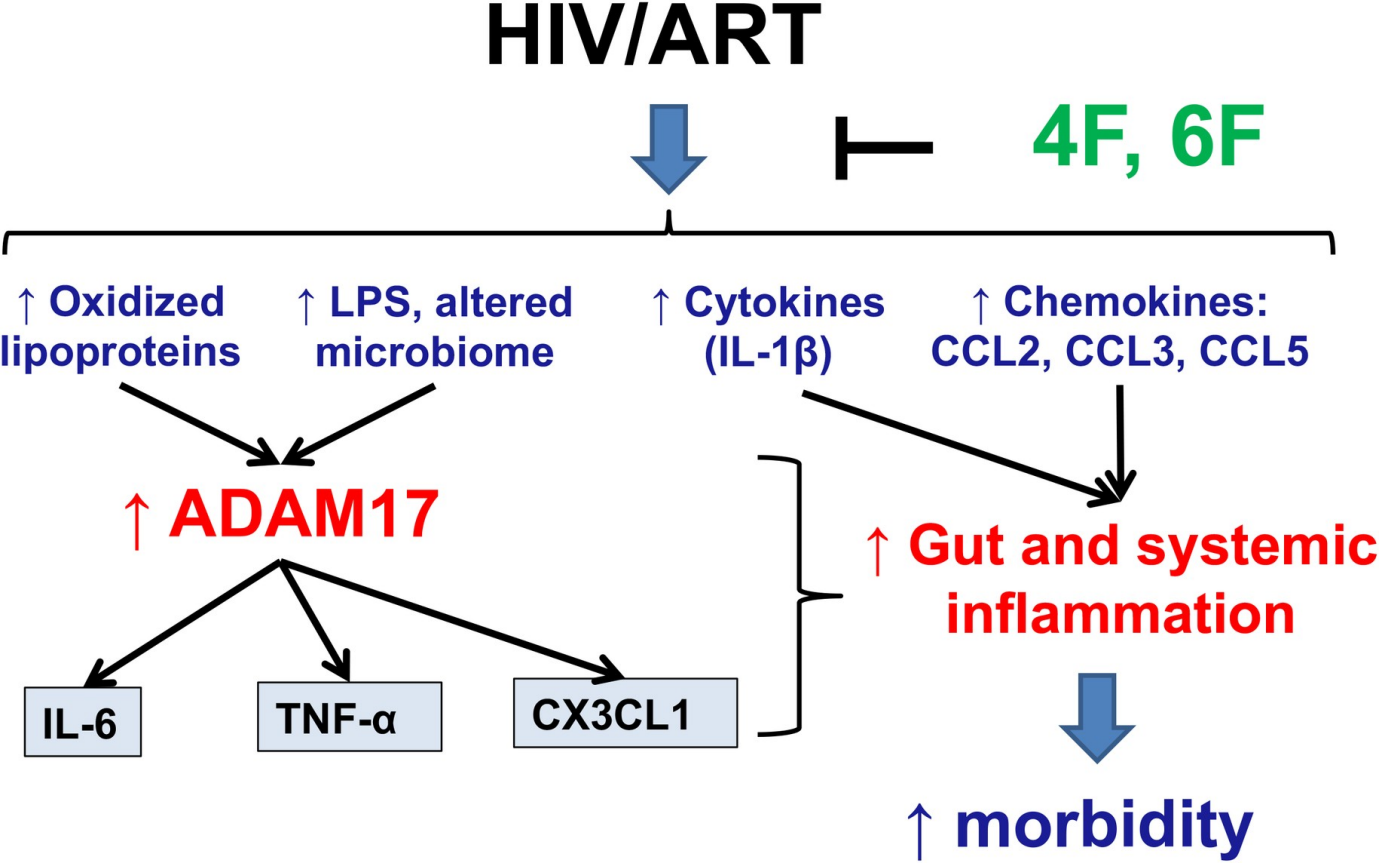
Overall Hypothesis. Systemic and intestinal levels of endotoxin (LPS) and oxidized lipoproteins (oxidized high- and low-density lipoproteins; HDLox and LDLox) are increased in chronic treated HIV despite potent antiretroviral therapy (ART). Oxidized lipoproteins and LPS increase protein levels of A disintegrin and metalloprotease 17 (ADAM17) in intestinal myeloid, epithelial and endothelial cells and protein levels of cytokines [interleukin (IL)-6, tumor necrosis factor alpha (TNF-α)] and chemokines [C-X3-C Motif Chemokine Ligand 1 (CX3CL1)], that are mediated by ADAM17 sheddase activity. ApoA-I mimetic peptides (4F, 6F) act mostly in the intestine and reduce levels of oxidized lipoproteins, LPS and microbial translocation, leading to reduction in levels of ADAM17 and secreted products of its sheddase activity (IL-6, TNF-α, CX3CL1). ApoA-I mimetic peptides (4F, 6F) also reduce other proinflammatory cytokines (IL-1β) and chemokines (CCL2, CCL3, CCL5) in blood and the intestine and ultimately reduce increased gut and systemic inflammation and morbidity in chronic treated HIV.

Oxidized lipoproteins play a major role in inflammation and immune activation in chronic treated HIV and are associated with sCD163, a major product of the ADAM17 sheddase [46]. Altered lipoproteins also trigger ADAM17 activity [50–52]. We have previously shown that Tg6F attenuates plasma and intestinal oxidized high- and low-density lipoproteins (HDLox and LDLox) in BLT mouse models of chronic treated HIV [24]. Thus, we further explored the associations of intestinal gut lipoproteins with gut protein levels of ADAM17. Murine intestinal protein levels of ADAM17 were positively associated with intestinal HDLox but not LDLox in HIV^+^ART^+^ TKO BLT mice (S1 Table). Protein cellular levels of ADAM17 did not correlate with plasma HDLox, in HIV^+^ART^+^Tg6F^+^ TKO BLT mice (S1 Table), consistent with our prior data that Tg6F is an intervention that reduces HDLox [24]. Gut explants of HIV^+^ and uninfected participants treated with HDLox (Fig 6) had higher intestinal protein levels of ADAM17 in myeloid CD33^+^CD45^+^ (Fig 6A and 6B) and CD324^+^CD45^-^ epithelial cells (Fig 6C and 6D) and intestinal TNF-α (Fig 6E) and CX3CL1 (Fig 6F) compared to vehicle controls (Fig 4A, 4B, 4C, 4D, 4E and 4F). Gut explants of HIV^+^ and uninfected participants treated with LDLox (Fig 7) had also higher intestinal protein levels of ADAM17 in myeloid CD33^+^CD45^+^ (Fig 7A and 7B) and CD324^+^CD45^-^ epithelial cells (Fig 7C and 7D) and intestinal TNF-α (Fig 7E) and CX3CL1 (Fig 7F) compared to vehicle controls (Fig 7A, 7B, 7C, 7D, 7E and 7F). Both 4F and 6F reduced ADAM17 in myeloid cells (Fig 6A and 6B) and epithelial cells (Fig 6C and 6D) and secretion of CX3CL1 (Fig 6F) from HDLox-treated gut explants from both uninfected and HIV^+^ participants. 4F tended (p = 0.089) to reduce secretion of TNF-α from HDLox-treated gut explants from uninfected participants (Fig 6E). 4F and 6F reduced secretion of TNF-α from HDLox-treated gut explants from HIV^+^ participants (Fig 6E). 4F and 6F also reduced ADAM17 in myeloid cells (Fig 7A and 7B) and epithelial cells (Fig 7C and 7D) and secretion of TNF-α (Fig 7E) and CX3CL1 (Fig 7F) from LDLox-treated gut explants from HIV^+^ participants. 4F also reduced ADAM17 in myeloid cells (Fig 7A and 7B) and epithelial cells (Fig 7C and 7D) from LDLox-treated gut explants from uninfected participants. 4F and 6F consistently and similarly reduced protein levels of ADAM17 in both myeloid and epithelial cells in gut explants from HIV^+^ participants with or without LPS (Fig 5), HDLox (Fig 6) and LDLox (Fig 7) treatments. Overall, our complementary BLT mouse and *ex vivo* human studies, demonstrated that apoA-I mimetic peptides attenuate proinflammatory effects of LPS and oxidized lipoproteins on ADAM17 and its sheddase activity that promote immune activation, systemic and intestinal inflammation in chronic treated HIV (Fig 8).

## Discussion

In independent preclinical models of chronic treated HIV, including BLT mice and human gut explants, we show that apoA-I mimetics consistently attenuated increased mediators of intestinal and systemic inflammation that are products of the ADAM17 sheddase activity (TNF-α, CX3CL1). ApoA-I mimetics also attenuated protein levels of ADAM17, that reflect signaling of both LPS [47–49] and bioactive lipids [50–52] in myeloid and intestinal cells and drive shedding of biomarkers of inflammation and morbidity such as TNF-α, IL-6, CX3CL1, sCD14, sCD163 and I-FABP [1,8,9,30] in chronic treated HIV. We also demonstrate that apoA-I mimetic peptides through favorable effects on bioactive lipids, dysfunctional HDL and LPS, that disrupt lipid rafts [53,54] and activate CX3CR1 and ADAM17[50–52], alters cytokines and immune cells in the intestinal wall. While low-level of HIV-1 replication may be seen in the BLT models, we previously demonstrated that Tg6F did not have an impact on viral replication [24] and the impact on biomarkers of inflammation was due to effect of Tg6F on LPS and oxidized lipoproteins. The component of the oral ApoA-I mimetic peptide Tg6F accounting for the beneficial effects on systemic inflammation is the 6F peptide, since control [11,13] had no effect on biomarkers of inflammation in chronic treated HIV. Given that Tg6F seems to work exclusively in the gut [11–14], our model (Fig 8) is proof-of-concept that therapeutic targeting of intestinal inflammation, intestinal LPS and bioactive lipids and gut barrier dysfunction may have a favorable impact on inflammation and immune activation that can complement ART to attenuate development of comorbidities like cardiovascular disease in chronic treated HIV.

Chronic treated HIV is a state of increased levels of bacterial translocation, and LPS signaling as evidenced by high levels of sCD14[1,8,9]. Activation of the TLR pathway by LPS is dependent on the co-localization of TLR4 and CD14 in lipid rafts that permit the activation of downstream signaling that culminate in NF-κB activation and the synthesis of pro-inflammatory cytokines [55]. Notably, ADAM17 is implicated in shedding of TLR4[47]. LPS has also been shown to directly upregulate *in vitro* ADAM17 levels in immune, epithelial and endothelial cells [48,49]. ADAM17 mediates further release of TNF-α that disrupts epithelial cell tight junctions [31] and triggers release of I-FABP [56]. In HIV^+^ART^+^ BLT mice protein levels of ADAM17 and plasma TNF-α and CX3CL1 positively associated with sCD14, suggesting a link between LPS signaling and ADAM17 protein levels in our BLT models of chronic treated HIV. Notably, in both BLT models, secretion of proinflammatory cytokines (TNF-α, IL-6) and chemokines (CX3CL1) and protein levels of ADAM17 were also increased in murine cells that are not infected by HIV suggesting a direct proinflammatory effect of murine LPS that was upregulated in BLT models of chronic treated HIV, independently of different ART regimens. These data are consistent with prior evidence that elevated LPS levels [2,8,46] are associated with biomarkers of increased morbidity in chronic HIV infection and trigger ADAM17 sheddase activity [25]. We also provide evidence based on gut explants of HIV-1 infected persons that apoA-I mimetics reduce LPS-induced expression of ADAM17 in intestinal epithelial and myeloid cells. These data are consistent with our prior findings that apoA-I mimetics attenuate chronic immune activation through reducing biomarkers of gut barrier dysfunction and bacterial translocation in preclinical models of chronic treated HIV [24]. Tg6F also reduced inflammation and small intestine enterocyte and plasma levels of LBP, CD14, TLR4 and MyD88[57] in murine models of disease [57,58]. Overall, our data are consistent with prior human studies that increased LPS and bacterial translocation are associated with systemic inflammation and immune activation in chronic treated HIV [1].

ART treated HIV infected individuals may have higher oxidative stress compared to HIV infected naïve or healthy subjects due to alterations in antioxidant systems [59,60]. During oxidative stress oxidized phospholipids acquire pleomorphic proinflammatory effects [61–65]. *In vivo* mechanistic animal models demonstrated that oxidized lipids are involved in the pathogenesis of inflammation and can be targeted therapeutically [66]. Several bioactive lipids interact with CX3CR1[36,67] that through NF-κB and CX3CL1 signaling contribute to inflammation and gut barrier function [6,32–35,44,45,68]. Bioactive lipids may also disrupt epithelial cell tight junctions either directly [69] or through lipid raft disruption [53,54] or TNF-α signaling [31] that collectively promote gut barrier dysfunction, intestinal inflammation, bacterial translocation and ultimately systemic inflammation. Consistent with our data in humans [46], plasma and intestinal HDLox levels rather than LDLox were associated with sCD163[24] and ADAM17 in HIV^+^ART^+^ BLT mice. While HDL is generally an anti-inflammatory lipoprotein during systemic inflammation it can be oxidized (HDLox) and becomes dysfunctional [15,70–72]. Our data are consistent with data from others that dysfunctional HDL and altered lipoproteins disrupt lipid rafts [54], contribute to increased protein levels of ADAM17[50] and activate ADAM17[50–52]. Oxidized LDL also carries oxidized lipids and like HDLox may also contribute to inflammation in HIV [46,73]. Notably, the associations between plasma and gut bioactive lipids with biomarkers of gut and systemic inflammation in the HIV^+^ART^+^EV^+^ group were attenuated in the HIV^+^ART^+^Tg6F^+^ group with an intervention that directly attenuates oxidized lipoproteins *in vivo* [24,58]. We confirmed these observations in preclinical *ex vivo* human gut explant models where both 4F and 6F directly attenuated ADAM17 and inflammatory responses triggered by oxidized lipoproteins.

Importantly, in all models we consistently found that apoA-I mimetics attenuated all major innate immunity biomarkers of inflammation that predict morbidity in chronic treated HIV including IL-1β, IL-6, TNF-α, CX3CL1, sCD14 and sCD163 [1–6,8,9,24,25]. In addition, both IL-1β[74] and LPS that are also associated with increased morbidity in chronic HIV infection [2,8] trigger ADAM17 sheddase activity [25]. Although ADAM17 levels have not been determined in chronic treated HIV, high levels of sCD163, a product of increased sheddase activity of ADAM17, in HIV infected ART-treated persons [1–6,8,9] and our preclinical data in HIV infected BLT mice, suggest increased protein levels of ADAM17 in treated HIV. Our data are consistent with previous evidence that ADAM17 is a potential therapeutic target for several inflammatory conditions [75–77] including viral infections (such as COVID-19) [78]. We propose the first therapeutic strategy that can target ADAM17 activity in chronic treated HIV.

Collectively, prior evidence [46–52,73] and our data suggest that increased LPS and oxidized lipoproteins drive increased ADAM17 activity as inducer of gut and systemic inflammation in chronic treated HIV. Importantly, in a prior clinical trial of 4F in humans, 4F was given parenterally and not orally [17] and did not improve HDL functionality [17]. Further work from our group demonstrated that apoA-I mimetics like 4F and 6F work primarily in the gut to inhibit gut inflammation and M/M activation [11–14], and oral apoA-I mimetics demonstrate therapeutic efficacy in several inflammatory diseases in animal models including cancer, cardiovascular and inflammatory bowel disease [11–14]. The safety and pharmacokinetics of oral apoA-I mimetic peptides have been tested in humans [16] and mice [11–14]. ApoA-I mimetic peptides seem to achieve higher concentration in the proximal small intestine [11–14]. This may explain how Tg6F that is not systemically absorbed [11,13,14], had a major favorable impact on systemic inflammation and immune activation in BLT models of chronic treated HIV.

Our study has limitations. Humanized mice do not fully recapitulate all aspects of HIV-1 immunopathogenesis due to presence of GVHD, suboptimal variable engraftment of human lymphocytes and macrophages, absence of fully functional secondary human lymphoid tissues and non-human microbiome [18–23]. Gut explants also only examine early effects of signaling. Thus, our findings may be explained by other underlying mechanisms or variables that were not studied in our preclinical models. Despite the limitations of humanized mouse models, we have shown the capacity of independent BLT mouse models to support HIV infection, express clinically relevant biomarkers of intestinal and systemic inflammation in the setting of chronic treated HIV and consistently respond to clinically relevant ART [24]. We excluded NSG mice with clinical GVHD and we also included studies with GVHD-resistant TKO BLT mice [21–23]. Gut explants also do not have limitations of other *ex vivo* intestinal models [79] and *ex vivo* addition of 4F in gut explants is the *only* preclinical approach in humans at this time. We did not include gut explants from HIV-1 infected women. Unlike other studies [80], we did not study the differential impact of hormones and sex-driven differences on the regulation of the gut inflammatory milieu in the setting of *ex vivo* use of apoA-I mimetics. We also did not study the differential impact of HIV-1 *per se*, antivirals, age, ethnicity and comorbidities on the regulation of the gut inflammatory milieu in the setting of *ex vivo* use of apoA-I mimetics. Further human studies are needed to determine the impact of apoA-I mimetic peptides on gut inflammation among different patient groups based on age, gender, ethnicity and comorbidities.

Overall, our robust experimental approach with independent preclinical *in vivo* and *ex vivo* experimental models and consistent findings provides important preclinical insight into the therapeutic impact of apoA-I mimetics on inflammation in chronic treated HIV. Our data support the hypothesis that apoA-I mimetic peptides attenuate a cycle of production of bioactive lipids and LPS that contribute to increased intestinal and systemic inflammation in chronic treated HIV.

## Materials and methods

### Ethics statement

This study was carried out in strict accordance with the recommendations in the Guide for the Care and Use of Laboratory Animals of the National Institutes of Health (” The Guide”), and was approved by the Institutional Animal Care and Use Committees of the University of California, Los Angeles, protocol # 1997–176. All individuals enrolled in the study provided written informed consent and the study was approved by the University of California Los Angeles Institutional Review Board [24].

### Materials

A concentrate of control transgenic tomatoes (EV) or a concentrate of transgenic tomatoes expressing the 6F peptide (Tg6F) were prepared and added to the diets at 0.06% by weight as described previously [11]. 4F and 6F peptides were synthesized as previously [14]. The antivirals Emtricitabine (FTC), Tenofovir Disoproxil Fumarate (TDF), Raltegravir (RAL), abacavir (ABC), dolutegravir (DOL), lamivudine (3TC), tenofovir alafenamide (TAF), FTC were either a generous gift from Gilead Sciences or were obtained from pharmacy. HIV p89.6 plasmid DNA was obtained from the NIH AIDS reagent Program. Murine chow diet with antibiotic was purchased from Envigo Teklad Diets. All other materials were purchased from commercially available sources and have been previously described in detail [24].

### Mice

The current study used murine biospecimens (blood and gut tissues) from a previously published study [24] to generate the data in this manuscript. As previously described [24] animal protocols were carried out in accordance with all federal, state, and local approved guidelines. The TKO or NSG BLT mice were generated and maintained as described [24]. A total of 5 (2 TKO and 3 NSG) independent cohorts of mice (each constructed from the same human donor tissues) were used for various experiments and were pooled for comparing mice that were uninfected (Group A: HIV^-^), infected and on potent ART (group B: HIV^+^, antivirals) and infected on potent ART and Tg6F (group C: HIV^+^, antivirals, Tg6F)(n = 10–20 mice per group) [24]. Given the variable engraftment of human immune cells and to better compare results among mice, we determined changes in human cytokines and chemokines among mice groups over 16 weeks of potent ART and we compared them to the uninfected group within each cohort (identical human donor) [24]. All the data from independent cohorts were then pooled together [24]. In the TKO BLT mice, Tg6F was given the day after the viral load was found to be suppressed while in the NSG BLT mice it was initiated 2 weeks before confirmation of suppression of viremia [24].

### HIV infection

Between 16 and 18 weeks, mice were challenged intraperitoneally with 500 ng p24 of HIV-1 dual-tropic 89.6 virus as described previously [24].

### ART treatment

As previously described [24], TKO C57 Bl6 mice were treated with ART consisted of combination medication of ABC/DOL/3TC (Triumeq) resuspended in the sweetened water gel formulation (Sucralose) as previously described [81]. As previously described [24], NSG BLT mice were treated with ART regimen consisting of TDF (8.75 mg/kg)/FTC (13 mg/kg)/RAL (17.5 mg/kg).

### Study participants

The current study used human biospecimens (gut tissues) from a previously published study [24]. HIV-seronegative (n = 10) and seropositive (n = 10) 50–60 years old participants were recruited in the Gastroenterology Unit of UCLA as previously described [24]. To avoid confounding effects from sex, race, inflammation (other than HIV) on M/M activation, we selected HIV^+^ white men, with no known clinical disease other than HIV or risk factors for clinical disease (e.g., metabolic syndrome, diabetes, dyslipidemia, use of lipid lowering medication). Six participants were on elvitegravir, cobicistat, emtricitabine (FTC), tenofovir alafenamide (TAF) and four participants were on bictegravir/FTC/TAF. All HIV infected persons had suppressed viremia (HIV-1 RNA <50 copies/ml), CD4^+^ T cells > 500 cells/mm^3^, duration of ART therapy between 3.2–6.5 years and nadir CD4^+^ T cell count > 250 cells/mm^3^. Ten healthy white men were also included as previously described [24].

### Gut explants

Biopsy specimens were obtained endoscopically from the rectosigmoid colon and gut mucosal explants were processed as previously described [24]. Briefly, 10–15 biopsies per participant were collected and cultured using RPMI 1640 medium with amphotericin B and piperacillin–tazobactam on absorbable gelatin sponge in tissue culture plates in a 37°C humidified incubator for 72 hours [24]. Triplicate biopsies were treated with media (control) or oxidized lipoproteins (HDLox, LDLox) at concentration 25 μg/ml [71,82] and/or with LPS at concentration 100 μg/ml [83] and/or with 4F, 6F at concentration 100 μg/ml [14] for 72 hours as previously described [14,71,82,83]. Supernatants were collected to measure secreted protein levels of cytokines (TNF-α) and chemokines (CX3CL1), that are mediated by ADAM17 sheddase activity. As previously described [24], the epithelial cells were isolated using DTT and EDTA while the remaining intestinal wall was further digested with the enzyme collagenase IV as previously described [84]. Protein levels of ADAM17 were determined in myeloid CD33^+^CD45^+^ and CD324^+^CD45^-^ epithelial cells of gut explants by flow cytometry.

### Oxidized lipoproteins

LDL was isolated from plasma of healthy subjects and the lipoproteins were oxidized by use of copper ions as previously [71,82]. HDL isolated from healthy participants was oxidized *in vitro* with 13(S)-HPODE as described [85]. Briefly, human HDL (5 μg HDL-cholesterol/ml) was incubated with 13(S)-HPODE (0.5 μg/ml), for 60 min (co-incubation). 13(S)-HPODE oxidized HDL and impaired HDL function in wild type C57BL/6J mice [85]. Thus, *in vitro* oxidation of HDL with 13(S)-HPODE is more physiologically relevant compared to copper ions that do not contribute to HDL dysfunction and oxidation of HDL *in vivo* [85].

### ApoA-I mimetics

For studies involving Tg6F, as previously described [24], the final diets contained 0.06% of Tg6F extract by weight and were administered for 12 weeks as previously described [11,12]. Triplicate biopsies were treated with 4F, 6F or sham peptide at concentration 100 μg/ml for 72 hours as previously described [14].

### Immunoassays

50–100 mg of gut tissue samples were placed in 2 ml screwcap dissociation tubes with ceramic beads (Precellys) and were mechanically dissociated in T-PER tissue protein extraction Reagent (Thermo Scientific) at power 5000 with two 20-second cycles as previously described [24]. Plasma and gut tissue human and murine cytokines (IL-1 beta, IL-6, h-IL-8, m-KC, IL-10, TNF-alpha), chemokines (CCL2, CCL3, CCL5, CX3CL1, CXCL10, h-IL-8/CXCL8) were determined using the human and murine magnetic Luminex performance assay kits according to the manufacturer instructions (R&D) and as previously described [24]. Murine CX3CL1/Fractalkine was determined by Quantikine ELISA as per manufacturer (R&D).

### Flow cytometry

For flow cytometry, single cell suspensions were incubated with the following antibodies: SYTOX Blue (Biolegend), FITC anti-human CD33 (clone P67.6), PE anti-human CD45 (clone HI30), PE/Dazzle 594 anti-mouse/human CD11b (clone M1/70), PerCP/Cyanine5.5 anti-mouse CD326 (Ep-CAM) clone G8.8, Alexa Fluor 700 anti-mouse CD45 (clone 30-F11), APC/Cyanine7 anti-mouse CD31 (Clone 390), APC/Cyanine7 anti-human CD326 (Ep-CAM) (clone 9C4), Human TACE/ADAM17 Ectodomain Alexa Fluor 647-conjugated Antibody (clone FAB9301R), anti-mouse ADAM17 Rabbit Monoclonal Antibody (clone JM10-35). All antibodies were obtained from Biolegend except for the anti-mouse ADAM17 Rabbit Monoclonal Antibody (clone JM10-35) (obtained from Thermo Fisher) and the Human TACE/ADAM17 Ectodomain Alexa Fluor 647-conjugated Antibody (clone FAB9301R) (obtained from R&D). For membrane antibody staining, intestinal cells were stained with surface markers. After 30 min, the cells were washed twice with fluorescence-activated cell sorting (FACS) buffer (PBS + 5% FBS). After a short spin, the cells were suspended in 200 μL of ice-cold PBS buffer and transferred to fresh tubes for FACS analysis. LSR Fortessa flow cytometer and FACSDiva software (BD Biosciences) were used for acquisition of flow cytometry data which were analyzed using FlowJo software. At least 1000 cells were acquired for each analysis, and each representative flow plot was repeated more than 3 times. Only live and singlet cells were chosen for analysis and gating (i.e., dead cells and aggregates were excluded).

### Statistics

*P* values less than 0.05 by Kruskal-Wallis or Mann-Whitney were considered significant. For all correlations, Spearman’s correlation coefficient was calculated. In the setting of exploratory approach we did not adjust for multiple comparisons since commonly-used multiple testing adjustment methods assume independence of tests, which in protein expression studies and in our explored pathways translates to a questionable assumption that all explored measures operate independently [86]. Instead, consistency between 2 independent BLT models, direction, and magnitude of the correlation coefficient in conjunction with the nominal p values were considered in order to help distinguish true and false-positive findings. All analyses were performed with Graphpad, version 7.0.

## Supporting information

S1 Dataset Includes the raw data used to make Figs 1A, 1B, 1C, 1d, 2A, 2B, 2C, 3B, 3D, 3F, 3H, 4B, 4D, 4E, 4F, 5B, 5D, 5E, 5F, 6B, 6D, 6E, 6F, 7B, 7D, 7E, 7F, S1A, S1B, S1C, S1D, S2A, S2B, S2C, S2D, S2E, S3A, S3B, S3C, S3D, S4, S5, S6B, S6C. (XLSX)

S1 Fig Biomarkers of systemic inflammation in NSG humanized model of chronic treated HIV. NSG humanized (BLT) mice were constructed and treated with control transgenic tomato concentrate (n = 11). Human and murine cytokines [interleukin (IL)-1β, IL-6, IL-8, IL-10, tumor necrosis factor alpha (TNF-α)] and chemokines [C-C Motif Chemokine Ligand 2 (CCL2), CCL3, CCL5, C-X3-C Motif Chemokine Ligand 1 (CX3CL1), C-X-C Motif Chemokine Ligand 10 (CXCL10)] were determined in plasma by Luminex immunoassays. **A-D.** Levels (pg/ml) of human (**A**) and murine (**B**) cytokines and human (**C**) and murine (**D**) chemokines in plasma from blood isolated from uninfected NSG (n = 11) BLT mice after 16 weeks of HIV infection in the HIV^+^ART ^+^ group. Data represent box and whiskers with minimum, median and maximum values (*n* = 11 mice per group). Datapoints represent mean of at least 2 experimental replicates per mouse. The Kruskal Wallis was used to compared >2 groups and the Mann-Whitney test was used to compare 2 groups (*p < 0.05, **p < 0.01, ***p < 0.001).
(TIF)

S2 Fig Biomarkers of systemic and gut inflammation in TKO humanized model of chronic treated HIV. TKO C57 humanized (BLT) mice were constructed and treated with control transgenic tomato concentrate. Blood and small intestine from each mouse were collected and plasma and protein lysates were prepared from uninfected TKO (n = 8) BLT mice after 16 weeks of HIV infection in the HIV^+^ART ^+^ group. Human and murine cytokines [interleukin (IL)-1β, IL-6, IL-8, IL-10, tumor necrosis factor alpha (TNF-α)] and chemokines [C-C Motif Chemokine Ligand 2 (CCL2), CCL3, CCL5, C-X3-C Motif Chemokine Ligand 1 (CX3CL1), C-X-C Motif Chemokine Ligand 10 (CXCL10)] were determined in plasma and gut tissue lysates by Luminex immunoassays. **A-D.** Levels (pg/ml) of human (**A**) and murine (**B**) cytokines and human (**C**) and murine (**D**) chemokines in plasma from blood isolated from uninfected TKO BLT mice. **E, F.** Levels (pg/μg of total protein) of human cytokines (**E**) and chemokines (**F**) in tissue lysates from gut isolated from uninfected TKO (n = 8) BLT mice after 16 weeks of HIV infection in the HIV^+^ART ^+^ group. Data represent box and whiskers with minimum, median and maximum values (*n* = 8 mice per group). Datapoints represent mean of at least 2 experimental replicates per mouse. The Kruskal Wallis was used to compared >2 groups and the Mann-Whitney test was used to compare 2 groups (*p < 0.05, **p < 0.01, ***p < 0.001).
(TIF)

S3 Fig Tg6F attenuates plasma murine cytokines and chemokines in BLT model of treated HIV infection. NSG (n = 51) and TKO (n = 37) humanized mice were constructed, infected with HIV and treated with antiretroviral therapy (ART), and control transgenic tomato concentrate and/or oral ApoA-I mimetic peptide 6F in the form of transgenic tomato (Tg6F). Murine cytokines [interleukin (IL)-1β, IL-6, KC/murine IL-8 homolog, IL-10, tumor necrosis factor alpha (TNF-α)] and chemokines [C-C Motif Chemokine Ligand 2 (CCL2), CCL3, CCL5, C-X3-C Motif Chemokine Ligand 1 (CX3CL1), C-X-C Motif Chemokine Ligand 10 (CXCL10)] were determined in plasma by Luminex immunoassays after 16 weeks of HIV infection in the HIV^+^ART ^+^ group. The mean value of each measurement in HIV^+^ART^+^ (red color) and HIV^+^ART^+^/Tg6F^+^ (blue color) mice BLT mice was expressed as fold to the mean value of each measurement in uninfected BLT mice (within the same cohort). **A.** Fold changes of murine cytokines in plasma of HIV^+^ART^+^ (n = 19) and HIV^+^ART^+^/Tg6F^+^ (n = 21) NSG mice compared to uninfected (n = 11) BLT mice. **B.** Fold changes of murine cytokines in plasma of HIV^+^ART^+^ (n = 14) and HIV^+^ART^+^/Tg6F^+^(n = 15) TKO mice compared to uninfected (n = 8) BLT mice. **C.** Fold changes of murine chemokines in plasma of HIV^+^ART^+^ (n = 19) and HIV^+^ART^+^/Tg6F^+^ (n = 21) NSG mice compared to uninfected (n = 11) BLT mice. **D.** Fold changes of murine chemokines in plasma of HIV^+^ART^+^ (n = 14) and HIV^+^ART^+^/Tg6F^+^(n = 15) TKO mice compared to uninfected (n = 8) BLT mice. Data represent box and whiskers with minimum, median and maximum values (*n* = 8–21 mice per group). Datapoints represent mean of at least 2 experimental replicates per mouse. The Kruskal Wallis was used to compared >2 groups and the Mann-Whitney test was used to compare 2 groups (*p < 0.05, **p < 0.01, ***p < 0.001). The asterisks in blue demonstrate the comparison relative to the uninfected group. **A.** Levels (pg/ml) of murine cytokines [interleukin (IL)-1β, IL-6, KC/murine IL8 homolog, IL-10, tumor necrosis factor alpha (TNF-α)] in plasma from uninfected NSG BLT mice after 16 weeks of HIV infection in the HIV^+^ART ^+^ group. **B.** Fold changes of murine cytokines in plasma of HIV^+^ART^+^ (red color) and HIV^+^ART^+^ /Tg6F^+^ (blue color) NSG mice compared to uninfected BLT mice. The mean value of each measurement was expressed as fold to the mean value of each measurement in uninfected BLT mice (within the same cohort). **C.** Levels (pg/ml) of murine cytokines in plasma from uninfected TKO BLT mice after 16 weeks of HIV infection in the HIV^+^ART ^+^ group. **D.** Fold changes of murine cytokines in plasma of HIV^+^ART^+^ (red color) and HIV^+^ART^+^/Tg6F^+^ (blue color) TKO mice compared to uninfected BLT mice. The mean value of each measurement was expressed as fold to the mean value of each measurement in uninfected BLT mice (within the same cohort). Data represent box and whiskers with minimum, median and maximum values (*n* = 8–22 mice per group). Datapoints represent mean of at least 2 experimental replicates per mouse. The Kruskal Wallis was used to compared >2 groups and the Mann-Whitney test was used to compare 2 groups (*p < 0.05, **p < 0.01, ***p < 0.001). The asterisks in blue demonstrate the comparison relative to the uninfected group.
(TIF)

S4 Fig Tg6F attenuates positive associations of gut protein levels of TNF-α, CX3CL1 with biomarkers of “gut barrier dysfunction” in humanized mouse model of chronic treated HIV infection. TKO C57 (n = 45) humanized mice were constructed, infected and treated with antiretroviral therapy (ART), and control transgenic tomato concentrate (EV) and/or oral ApoA-I mimetic peptide 6F in the form of transgenic tomato (Tg6F). Murine TNF-α and CX3CL1 were determined by Luminex immunoassays as in Materials and Methods. Plasma murine sCD14 and I-FABP are biomarkers of “leaky gut” that predict morbidity in chronic treated HIV and were determined by ELISAs. Scatter plots of gut protein levels of murine TNF-α and CX3CL1 (x axis) against biomarkers of “leaky gut” (y axis) are shown for the ART-treated groups and the ART+Tg6F groups (*n* = 8–22 mice per group). The Spearman correlation coefficient was used for all correlations between i) plasma sCD14 with protein levels of gut TNF-α, **(A, C),** gut m-CX3CL1 (**B, D),** in HIV+ART+ treated mice (**A, B**), and HIV+ART+Tg6F+ treated mice (**C, D)**; ii) plasma I-FABP with protein levels of gut TNF-α (**E, G),** gut m-CX3CL1, (**F, H),** in HIV+ART+ treated mice, (**E, F**) and HIV+ART+Tg6F+ treated mice (**G, H**).
(TIF)

S5 Fig A-I. Gating strategy in flow cytometry experiments in intestinal cells of humanized mice. TKO C57 (n = 45) humanized (BLT) mice were constructed, infected with HIV and treated with antiretroviral therapy (ART) and control transgenic tomato concentrate and/or oral ApoA-I mimetic peptide 6F in the form of transgenic tomato (Tg6F). Single cell suspension from small intestine was prepared and was used for flow cytometry in intestinal cells. Fluorescence intensity of a positive cell population was compared to a negative cell population (fluorescence minus one negative control for staining) (ΔMFI). Representative flow cytometry data from gut single cell suspension of TKO BLT mice: A. 1) Single cells (SSC); B. ➔ 2) Single cells (FSC); C. 3) Cells (FSC/SSC) to exclude debris; D. ➔ 4) Viable cells were gated as negative stain for the SYTOX Blue dead cell stain; E. 5) Human immune cells were gated as hCD45^+^ on gate 4. F. 6) Human myeloid cells were gated as hCD33+ on hCD45+ cells on gate 5; G. 7) Murine immune cells were gated as mCD45+ on gate 4. H. 8) Murine myeloid cells were gated as hCD11b^+^ on mCD45^+^ cells on gate 7; I. 9) Murine epithelial cells were gated as m-CD326^+^ on mCD45- cells on gate 7. 10) Murine endothelial cells were gated as mCD31^+^ on m-CD326^-^, m-CD45- cells on gate 9 (not shown).
(TIF)

S6 Fig Tg6F attenuates increases in protein levels of CXCR3 in human gut macrophages in treated HIV infection. Single cell suspension from gut was prepared and cellular membrane protein levels of human CXCR3 (h-CXCR3) in human CD33^+^CD45^+^ myeloid immune cells were determined by flow cytometry. **A.** Representative data of median fluorescence intensity (MFI) of a positive cell population compared to a negative cell population (fluorescence minus one negative control for staining shown in light filled grey histogram)] of h-CXCR3 in human h-CD33^+^h-CD45^+^ myeloid immune cells are shown**. B.** Summary of data (ΔMFI h-CXCR3) for **(A). C.** Summary of data for ΔMFI m-CXCR3 in murine m-CD11b^+^CD45^+^ myeloid immune cells. Data represent box and whiskers with minimum, median and maximum values (*n* = 8–22 mice per group). The Mann-Whitney test was used to compare 2 groups (*p < 0.05, **p < 0.01, ***p < 0.001).
(TIF)

S1 Table Associations of protein levels (% of positive cells and ΔMFI) of ADAM-17 in human CD33+ myeloid cells and murine CD326+ epithelial cells with gut oxidized lipoproteins (HDLox, LDLox), plasma m-IFABP, human and murine sCD14 and sCD163 levels in HIV+/ART treated TKO BLT mice (n = 14) and HIV+/ART/Tg6F (n = 15) treated TKO BLT mice. Spearman correlation coefficient (r) is shown for all associations between pairs. Statistically significant associations (p<0.05) are indicated in bold. Trends for associations (0.05<p<0.10) are underlined in italics.
(PDF)

## References

[1] Lederman MM, Funderburg NT, Sekaly RP, Klatt NR, Hunt PW. Residual immune dysregulation syndrome in treated HIV infection. Adv Immunol. 2013;119:51–83. doi: 10.1016/B978-0-12-407707-2.00002-3 ; PubMed Central PMCID: PMC4126613. 23886064 PMC4126613

[2] Hsue PY, Li D, Ma Y, Ishai A, Manion M, Nahrendorf M, et al. IL-1beta Inhibition Reduces Atherosclerotic Inflammation in HIV Infection. J Am Coll Cardiol. 2018;72(22):2809–11. Epub 2018/12/01. doi: 10.1016/j.jacc.2018.09.038 . 30497570 PMC8723182

[3] Margolick JB, Bream JH, Martinez-Maza O, Lopez J, Li X, Phair JP, et al. Frailty and Circulating Markers of Inflammation in HIV+ and HIV- Men in the Multicenter AIDS Cohort Study. J Acquir Immune Defic Syndr. 2017;74(4):407–17. Epub 2017/02/23. doi: 10.1097/QAI.0000000000001261 ; PubMed Central PMCID: PMC5365031. 28225718 PMC5365031

[4] Hsu DC, Ma YF, Hur S, Li D, Rupert A, Scherzer R, et al. Plasma IL-6 levels are independently associated with atherosclerosis and mortality in HIV-infected individuals on suppressive antiretroviral therapy. AIDS. 2016;30(13):2065–74. Epub 2016/05/14. doi: 10.1097/QAD.0000000000001149 ; PubMed Central PMCID: PMC5586221. 27177313 PMC5586221

[5] Pasquereau S, Kumar A, Herbein G. Targeting TNF and TNF Receptor Pathway in HIV-1 Infection: from Immune Activation to Viral Reservoirs. Viruses. 2017;9(4). Epub 2017/03/31. doi: 10.3390/v9040064 ; PubMed Central PMCID: PMC5408670. 28358311 PMC5408670

[6] Cotter R, Williams C, Ryan L, Erichsen D, Lopez A, Peng H, et al. Fractalkine (CX3CL1) and brain inflammation: Implications for HIV-1-associated dementia. J Neurovirol. 2002;8(6):585–98. Epub 2002/12/12. doi: 10.1080/13550280290100950 . 12476352

[7] Funderburg NT, Zidar DA, Shive C, Lioi A, Mudd J, Musselwhite LW, et al. Shared monocyte subset phenotypes in HIV-1 infection and in uninfected subjects with acute coronary syndrome. Blood. 2012;120(23):4599–608. doi: 10.1182/blood-2012-05-433946 23065151 PMC3512236

[8] Sandler NG, Wand H, Roque A, Law M, Nason MC, Nixon DE, et al. Plasma levels of soluble CD14 independently predict mortality in HIV infection. J Infect Dis. 2011;203(6):780–90. doi: 10.1093/infdis/jiq118 21252259 PMC3071127

[9] Knudsen TB, Ertner G, Petersen J, Moller HJ, Moestrup SK, Eugen-Olsen J, et al. Plasma Soluble CD163 Level Independently Predicts All-Cause Mortality in HIV-1-Infected Individuals. J Infect Dis. 2016;214(8):1198–204. doi: 10.1093/infdis/jiw263 . 27354366

[10] Navab M, Shechter I, Anantharamaiah GM, Reddy ST, Van Lenten BJ, Fogelman AM. Structure and function of HDL mimetics. ArteriosclerThrombVascBiol. 2010;30(2):164–8. doi: 10.1161/ATVBAHA.109.187518 19608977 PMC2860541

[11] Chattopadhyay A, Navab M, Hough G, Gao F, Meriwether D, Grijalva V, et al. A novel approach to oral apoA-I mimetic therapy. J Lipid Res. 2013;54(4):995–1010. doi: 10.1194/jlr.M033555 ; PubMed Central PMCID: PMC3606004. 23378594 PMC3606004

[12] Chattopadhyay A, Grijalva V, Hough G, Su F, Mukherjee P, Farias-Eisner R, et al. Efficacy of tomato concentrates in mouse models of dyslipidemia and cancer. Pharmacol Res Perspect. 2015;3(4):e00154. doi: 10.1002/prp2.154 ; PubMed Central PMCID: PMC4492730. 26171234 PMC4492730

[13] Chattopadhyay A, Yang X, Mukherjee P, Sulaiman D, Fogelman HR, Grijalva V, et al. Treating the Intestine with Oral ApoA-I Mimetic Tg6F Reduces Tumor Burden in Mouse Models of Metastatic Lung Cancer. Sci Rep. 2018;8(1):9032. doi: 10.1038/s41598-018-26755-0 ; PubMed Central PMCID: PMC5998131. 29899427 PMC5998131

[14] Meriwether D, Sulaiman D, Volpe C, Dorfman A, Grijalva V, Dorreh N, et al. Apolipoprotein A-I mimetics mitigate intestinal inflammation in COX2-dependent inflammatory bowel disease model. J Clin Invest. 2019;130(9):3670–85. Epub 2019/06/12. doi: 10.1172/JCI123700 ; PubMed Central PMCID: PMC6715371. 31184596 PMC6715371

[15] Kelesidis T, Yang OO, Currier JS, Navab K, Fogelman AM, Navab M. HIV-1 infected patients with suppressed plasma viremia on treatment have pro-inflammatory HDL. Lipids Health Dis. 2011;10:35. Epub 2011/02/25. doi: 10.1186/1476-511X-10-35 ; PubMed Central PMCID: PMC3049748. 21345230 PMC3049748

[16] Bloedon LT, Dunbar R, Duffy D, Pinell-Salles P, Norris R, DeGroot BJ, et al. Safety, pharmacokinetics, and pharmacodynamics of oral apoA-I mimetic peptide D-4F in high-risk cardiovascular patients. JLipid Res. 2008;49(6):1344–52. 18323573 10.1194/jlr.P800003-JLR200 PMC2386905

[17] Watson CE, Weissbach N, Kjems L, Ayalasomayajula S, Zhang Y, Chang I, et al. Treatment of Patients with Cardiovascular Disease with L-4F, an Apo-A1 mimetic, Did Not Improve Select Biomarkers of HDL Function. J Lipid Res. 2011;52(2):361–73. doi: 10.1194/jlr.M011098 21068008 PMC3023557

[18] Melkus MW, Estes JD, Padgett-Thomas A, Gatlin J, Denton PW, Othieno FA, et al. Humanized mice mount specific adaptive and innate immune responses to EBV and TSST-1. Nat Med. 2006;12(11):1316–22. Epub 2006/10/24. nm1431 [pii] doi: 10.1038/nm1431 . 17057712

[19] Zhen A, Rezek V, Youn C, Lam B, Chang N, Rick J, et al. Targeting type I interferon-mediated activation restores immune function in chronic HIV infection. J Clin Invest. 2017;127(1):260–8. Epub 2016/12/13. doi: 10.1172/JCI89488 ; PubMed Central PMCID: PMC5199686. 27941243 PMC5199686

[20] Hofer U, Schlaepfer E, Baenziger S, Nischang M, Regenass S, Schwendener R, et al. Inadequate clearance of translocated bacterial products in HIV-infected humanized mice. PLoS Pathog. 2010;6(4):e1000867. Epub 2010/05/06. doi: 10.1371/journal.ppat.1000867 ; PubMed Central PMCID: PMC2861703. 20442871 PMC2861703

[21] Lavender KJ, Messer RJ, Race B, Hasenkrug KJ. Production of bone marrow, liver, thymus (BLT) humanized mice on the C57BL/6 Rag2(-/-)gammac(-/-)CD47(-/-) background. J Immunol Methods. 2014;407:127–34. Epub 2014/04/29. doi: 10.1016/j.jim.2014.04.008 ; PubMed Central PMCID: PMC4060441. 24769067 PMC4060441

[22] Lavender KJ, Pace C, Sutter K, Messer RJ, Pouncey DL, Cummins NW, et al. An advanced BLT-humanized mouse model for extended HIV-1 cure studies. AIDS. 2018;32(1):1–10. Epub 2017/11/08. doi: 10.1097/QAD.0000000000001674 ; PubMed Central PMCID: PMC5718929. 29112072 PMC5718929

[23] Lavender KJ, Pang WW, Messer RJ, Duley AK, Race B, Phillips K, et al. BLT-humanized C57BL/6 Rag2-/-gammac-/-CD47-/- mice are resistant to GVHD and develop B- and T-cell immunity to HIV infection. Blood. 2013;122(25):4013–20. Epub 2013/09/12. doi: 10.1182/blood-2013-06-506949 ; PubMed Central PMCID: PMC3862274. 24021673 PMC3862274

[24] Mu W, Sharma M, Heymans R, Ritou E, Rezek V, Hamid P, et al. Apolipoprotein A-I mimetics attenuate macrophage activation in chronic treated HIV. AIDS. 2021;35(4):543–53. Epub 2020/12/12. doi: 10.1097/QAD.0000000000002785 ; PubMed Central PMCID: PMC8010648. 33306550 PMC8010648

[25] Gooz M. ADAM-17: the enzyme that does it all. Crit Rev Biochem Mol Biol. 2010;45(2):146–69. doi: 10.3109/10409231003628015 ; PubMed Central PMCID: PMC2841225. 20184396 PMC2841225

[26] Kelesidis T, Tran TT, Stein JH, Brown TT, Moser C, Ribaudo HJ, et al. Changes in Inflammation and Immune Activation With Atazanavir-, Raltegravir-, Darunavir-Based Initial Antiviral Therapy: ACTG 5260s. Clin Infect Dis. 2015;61(4):651–60. doi: 10.1093/cid/civ327 ; PubMed Central PMCID: PMC4542595. 25904376 PMC4542595

[27] Smythies LE, White CR, Maheshwari A, Palgunachari MN, Anantharamaiah GM, Chaddha M, et al. Apolipoprotein A-I mimetic 4F alters the function of human monocyte-derived macrophages. Am J Physiol Cell Physiol. 2010;298(6):C1538–48. doi: 10.1152/ajpcell.00467.2009 ; PubMed Central PMCID: PMC2889631. 20219948 PMC2889631

[28] Van Lenten BJ, Wagner AC, Jung CL, Ruchala P, Waring AJ, Lehrer RI, et al. Anti-inflammatory apoA-I-mimetic peptides bind oxidized lipids with much higher affinity than human apoA-I. J Lipid Res. 2008;49(11):2302–11. doi: 10.1194/jlr.M800075-JLR200 18621920 PMC2563211

[29] Navab M, Anantharamaiah GM, Reddy ST, Fogelman AM. Apolipoprotein A-I mimetic peptides and their role in atherosclerosis prevention. NatClinPractCardiovascMed. 2006;3(10):540–7. doi: 10.1038/ncpcardio0661 16990839

[30] Anzinger JJ, Butterfield TR, Angelovich TA, Crowe SM, Palmer CS. Monocytes as regulators of inflammation and HIV-related comorbidities during cART. J Immunol Res. 2014;2014:569819. doi: 10.1155/2014/569819 ; PubMed Central PMCID: PMC4082935. 25025081 PMC4082935

[31] Freour T, Jarry A, Bach-Ngohou K, Dejoie T, Bou-Hanna C, Denis MG, et al. TACE inhibition amplifies TNF-alpha-mediated colonic epithelial barrier disruption. Int J Mol Med. 2009;23(1):41–8. . 19082505

[32] Jones BA, Beamer M, Ahmed S. Fractalkine/CX3CL1: a potential new target for inflammatory diseases. Mol Interv. 2010;10(5):263–70. Epub 2010/11/04. doi: 10.1124/mi.10.5.3 ; PubMed Central PMCID: PMC3002219. 21045240 PMC3002219

[33] Brand S, Sakaguchi T, Gu X, Colgan SP, Reinecker HC. Fractalkine-mediated signals regulate cell-survival and immune-modulatory responses in intestinal epithelial cells. Gastroenterology. 2002;122(1):166–77. Epub 2002/01/10. doi: 10.1053/gast.2002.30329 . 11781291

[34] Faure S, Meyer L, Costagliola D, Vaneensberghe C, Genin E, Autran B, et al. Rapid progression to AIDS in HIV+ individuals with a structural variant of the chemokine receptor CX3CR1. Science. 2000;287(5461):2274–7. Epub 2000/03/24. doi: 10.1126/science.287.5461.2274 . 10731151

[35] Foussat A, Bouchet-Delbos L, Berrebi D, Durand-Gasselin I, Coulomb-L’Hermine A, Krzysiek R, et al. Deregulation of the expression of the fractalkine/fractalkine receptor complex in HIV-1-infected patients. Blood. 2001;98(6):1678–86. Epub 2001/09/06. doi: 10.1182/blood.v98.6.1678 . 11535497

[36] Barlic J, Zhang Y, Foley JF, Murphy PM. Oxidized lipid-driven chemokine receptor switch, CCR2 to CX3CR1, mediates adhesion of human macrophages to coronary artery smooth muscle cells through a peroxisome proliferator-activated receptor gamma-dependent pathway. Circulation. 2006;114(8):807–19. doi: 10.1161/CIRCULATIONAHA.105.602359 . 16908772

[37] Bernardo D, Marin AC, Fernandez-Tome S, Montalban-Arques A, Carrasco A, Tristan E, et al. Human intestinal pro-inflammatory CD11c(high)CCR2(+)CX3CR1(+) macrophages, but not their tolerogenic CD11c(-)CCR2(-)CX3CR1(-) counterparts, are expanded in inflammatory bowel disease. Mucosal Immunol. 2018;11(4):1114–26. Epub 2018/05/11. doi: 10.1038/s41385-018-0030-7 . 29743615

[38] Nazli A, Kafka JK, Ferreira VH, Anipindi V, Mueller K, Osborne BJ, et al. HIV-1 gp120 induces TLR2- and TLR4-mediated innate immune activation in human female genital epithelium. J Immunol. 2013;191(8):4246–58. doi: 10.4049/jimmunol.1301482 . 24043886

[39] Kirkegaard T, Pedersen G, Saermark T, Brynskov J. Tumour necrosis factor-alpha converting enzyme (TACE) activity in human colonic epithelial cells. Clin Exp Immunol. 2004;135(1):146–53. doi: 10.1111/j.1365-2249.2004.02348.x ; PubMed Central PMCID: PMC1808921. 14678276 PMC1808921

[40] Cario E, Podolsky DK. Differential alteration in intestinal epithelial cell expression of toll-like receptor 3 (TLR3) and TLR4 in inflammatory bowel disease. Infect Immun. 2000;68(12):7010–7. doi: 10.1128/IAI.68.12.7010-7017.2000 ; PubMed Central PMCID: PMC97811. 11083826 PMC97811

[41] Ding Z, Liu S, Wang X, Khaidakov M, Dai Y, Deng X, et al. Lectin-like ox-LDL receptor-1 (LOX-1)-Toll-like receptor 4 (TLR4) interaction and autophagy in CATH.a differentiated cells exposed to angiotensin II. Mol Neurobiol. 2015;51(2):623–32. doi: 10.1007/s12035-014-8756-z . 24902807

[42] Zhao W, Ma G, Chen X. Lipopolysaccharide induced LOX-1 expression via TLR4/MyD88/ROS activated p38MAPK-NF-kappaB pathway. Vascul Pharmacol. 2014;63(3):162–72. doi: 10.1016/j.vph.2014.06.008 . 25135647

[43] Feng Y, Cai ZR, Tang Y, Hu G, Lu J, He D, et al. TLR4/NF-kappaB signaling pathway-mediated and oxLDL-induced up-regulation of LOX-1, MCP-1, and VCAM-1 expressions in human umbilical vein endothelial cells. Genet Mol Res. 2014;13(1):680–95. doi: 10.4238/2014.January.28.13 . 24615033

[44] Marsche G, Levak-Frank S, Quehenberger O, Heller R, Sattler W, Malle E. Identification of the human analog of SR-BI and LOX-1 as receptors for hypochlorite-modified high density lipoprotein on human umbilical venous endothelial cells. FASEB J. 2001;15(6):1095–7. doi: 10.1096/fj.00-0532fje 11292679

[45] Mitra S, Goyal T, Mehta JL. Oxidized LDL, LOX-1 and atherosclerosis. Cardiovasc Drugs Ther. 2011;25(5):419–29. doi: 10.1007/s10557-011-6341-5 . 21947818

[46] Kelesidis T, Jackson N, McComsey GA, Wang X, Elashoff D, Dube MP, et al. Oxidized lipoproteins are associated with markers of inflammation and immune activation in HIV-1 infection. AIDS. 2016;30(17):2625–33. doi: 10.1097/QAD.0000000000001238 ; PubMed Central PMCID: PMC5083154. 27603288 PMC5083154

[47] Yang WS, Kim JJ, Lee MJ, Lee EK, Park SK. ADAM17-Mediated Ectodomain Shedding of Toll-Like Receptor 4 as a Negative Feedback Regulation in Lipopolysaccharide-Activated Aortic Endothelial Cells. Cell Physiol Biochem. 2018;45(5):1851–62. Epub 2018/03/07. doi: 10.1159/000487876 . 29510400

[48] Arndt PG, Strahan B, Wang Y, Long C, Horiuchi K, Walcheck B. Leukocyte ADAM17 regulates acute pulmonary inflammation. PLoS One. 2011;6(5):e19938. Epub 2011/05/24. doi: 10.1371/journal.pone.0019938 ; PubMed Central PMCID: PMC3095620. 21603616 PMC3095620

[49] Dreymueller D, Martin C, Kogel T, Pruessmeyer J, Hess FM, Horiuchi K, et al. Lung endothelial ADAM17 regulates the acute inflammatory response to lipopolysaccharide. EMBO Mol Med. 2012;4(5):412–23. Epub 2012/03/01. doi: 10.1002/emmm.201200217 ; PubMed Central PMCID: PMC3403298. 22367719 PMC3403298

[50] Carnuta MG, Stancu CS, Toma L, Sanda GM, Niculescu LS, Deleanu M, et al. Dysfunctional high-density lipoproteins have distinct composition, diminished anti-inflammatory potential and discriminate acute coronary syndrome from stable coronary artery disease patients. Sci Rep. 2017;7(1):7295. doi: 10.1038/s41598-017-07821-5 ; PubMed Central PMCID: PMC5544737. 28779156 PMC5544737

[51] Matthews V, Schuster B, Schutze S, Bussmeyer I, Ludwig A, Hundhausen C, et al. Cellular cholesterol depletion triggers shedding of the human interleukin-6 receptor by ADAM10 and ADAM17 (TACE). J Biol Chem. 2003;278(40):38829–39. Epub 2003/07/02. doi: 10.1074/jbc.M210584200 . 12832423

[52] Tellier E, Canault M, Poggi M, Bonardo B, Nicolay A, Alessi MC, et al. HDLs activate ADAM17-dependent shedding. J Cell Physiol. 2008;214(3):687–93. doi: 10.1002/jcp.21265 . 17786981

[53] Chen ML, Ge Z, Fox JG, Schauer DB. Disruption of tight junctions and induction of proinflammatory cytokine responses in colonic epithelial cells by Campylobacter jejuni. Infect Immun. 2006;74(12):6581–9. doi: 10.1128/IAI.00958-06 ; PubMed Central PMCID: PMC1698078. 17015453 PMC1698078

[54] Levitan I, Shentu TP. Impact of oxLDL on Cholesterol-Rich Membrane Rafts. J Lipids. 2011;2011:730209. doi: 10.1155/2011/730209 21490811 PMC3066652

[55] Triantafilou M, Gamper FG, Haston RM, Mouratis MA, Morath S, Hartung T, et al. Membrane sorting of toll-like receptor (TLR)-2/6 and TLR2/1 heterodimers at the cell surface determines heterotypic associations with CD36 and intracellular targeting. J Biol Chem. 2006;281(41):31002–11. Epub 2006/08/02. doi: 10.1074/jbc.M602794200 . 16880211

[56] Marchetti G, Tincati C, Silvestri G. Microbial translocation in the pathogenesis of HIV infection and AIDS. Clin Microbiol Rev. 2013;26(1):2–18. Epub 2013/01/09. doi: 10.1128/CMR.00050-12 ; PubMed Central PMCID: PMC3553668. 23297256 PMC3553668

[57] Mukherjee P, Hough G, Chattopadhyay A, Grijalva V, O’Connor EI, Meriwether D, et al. Role of enterocyte stearoyl-Co-A desaturase-1 in LDLR-null mice. J Lipid Res. 2018;59(10):1818–40. Epub 2018/08/25. doi: 10.1194/jlr.M083527 ; PubMed Central PMCID: PMC6168294. 30139760 PMC6168294

[58] Chattopadhyay A, Navab M, Hough G, Grijalva V, Mukherjee P, Fogelman HR, et al. Tg6F ameliorates the increase in oxidized phospholipids in the jejunum of mice fed unsaturated LysoPC or WD. J Lipid Res. 2016;57(5):832–47. Epub 2016/03/12. doi: 10.1194/jlr.M064352 ; PubMed Central PMCID: PMC4847630. 26965826 PMC4847630

[59] Sharma B. Oxidative stress in HIV patients receiving antiretroviral therapy. Curr HIV Res. 2014;12(1):13–21. doi: 10.2174/1570162x12666140402100959 . 24694264

[60] Mandas A, Iorio EL, Congiu MG, Balestrieri C, Mereu A, Cau D, et al. Oxidative imbalance in HIV-1 infected patients treated with antiretroviral therapy. J Biomed Biotechnol. 2009;2009:749575. doi: 10.1155/2009/749575 ; PubMed Central PMCID: PMC2768042. 19884983 PMC2768042

[61] Tsimikas S, Miller YI. Oxidative modification of lipoproteins: mechanisms, role in inflammation and potential clinical applications in cardiovascular disease. Curr Pharm Des. 2011;17(1):27–37. Epub 2011/01/14. doi: 10.2174/138161211795049831 . 21226665

[62] Laczik R, Szodoray P, Veres K, Lakos G, Sipka S, Szegedi G, et al. Oxidized LDL induces in vitro lymphocyte activation in antiphospholipid syndrome. Autoimmunity. 2010;43(4):334–9. Epub 2010/03/02. doi: 10.3109/08916930903540440 . 20187701

[63] Park KH, Cho KH. High-density lipoprotein (HDL) from elderly and reconstituted HDL containing glycated apolipoproteins A-I share proatherosclerotic and prosenescent properties with increased cholesterol influx. J Gerontol A Biol Sci Med Sci. 2011;66(5):511–20. doi: 10.1093/gerona/glr016 . 21415260

[64] Park KH, Shin DG, Cho KH. Dysfunctional lipoproteins from young smokers exacerbate cellular senescence and atherogenesis with smaller particle size and severe oxidation and glycation. Toxicol Sci. 2014;140(1):16–25. doi: 10.1093/toxsci/kfu076 . 24798380

[65] Hansson GK, Hermansson A. The immune system in atherosclerosis. Nat Immunol. 2011;12(3):204–12. Epub 2011/02/16. doi: 10.1038/ni.2001 . 21321594

[66] Que X, Hung MY, Yeang C, Gonen A, Prohaska TA, Sun X, et al. Oxidized phospholipids are proinflammatory and proatherogenic in hypercholesterolaemic mice. Nature. 2018;558(7709):301–6. doi: 10.1038/s41586-018-0198-8 ; PubMed Central PMCID: PMC6033669. 29875409 PMC6033669

[67] Rolin J, Maghazachi AA. Implications of chemokines, chemokine receptors, and inflammatory lipids in atherosclerosis. J Leukoc Biol. 2014;95(4):575–85. Epub 2014/02/05. doi: 10.1189/jlb.1113571 . 24493826

[68] Eichhorn B, Muller G, Leuner A, Sawamura T, Ravens U, Morawietz H. Impaired vascular function in small resistance arteries of LOX-1 overexpressing mice on high-fat diet. Cardiovasc Res. 2009;82(3):493–502. Epub 2009/03/18. doi: 10.1093/cvr/cvp089 . 19289377

[69] Chen-Quay SC, Eiting KT, Li AW, Lamharzi N, Quay SC. Identification of tight junction modulating lipids. J Pharm Sci. 2009;98(2):606–19. doi: 10.1002/jps.21462 . 18563833

[70] Navab M, Reddy ST, Van Lenten BJ, Fogelman AM. HDL and cardiovascular disease: atherogenic and atheroprotective mechanisms. Nat Rev Cardiol. 2011;8(4):222–32. Epub 2011/02/10. doi: 10.1038/nrcardio.2010.222 . 21304474

[71] Kelesidis T, Currier JS, Huynh D, Meriwether D, Charles-Schoeman C, Reddy ST, et al. A biochemical fluorometric method for assessing the oxidative properties of HDL. J Lipid Res. 2011;52(12):2341–51. Epub 2011/10/01. doi: 10.1194/jlr.D018937 ; PubMed Central PMCID: PMC3220300. 21957198 PMC3220300

[72] Kelesidis T, Roberts CK, Huynh D, Martinez-Maza O, Currier JS, Reddy ST, et al. A high throughput biochemical fluorometric method for measuring lipid peroxidation in HDL. PLoS One. 2014;9(11):e111716. doi: 10.1371/journal.pone.0111716 ; PubMed Central PMCID: PMC4219769. 25368900 PMC4219769

[73] Zidar DA, Juchnowski S, Ferrari B, Clagett B, Pilch-Cooper HA, Rose S, et al. Oxidized LDL Levels Are Increased in HIV Infection and May Drive Monocyte Activation. J Acquir Immune Defic Syndr. 2015;69(2):154–60. doi: 10.1097/QAI.0000000000000566 ; PubMed Central PMCID: PMC4446174. 25647528 PMC4446174

[74] Hall KC, Blobel CP. Interleukin-1 stimulates ADAM17 through a mechanism independent of its cytoplasmic domain or phosphorylation at threonine 735. PLoS One. 2012;7(2):e31600. Epub 2012/03/03. doi: 10.1371/journal.pone.0031600 ; PubMed Central PMCID: PMC3288042. 22384041 PMC3288042

[75] Li R, Uttarwar L, Gao B, Charbonneau M, Shi Y, Chan JS, et al. High Glucose Up-regulates ADAM17 through HIF-1alpha in Mesangial Cells. J Biol Chem. 2015;290(35):21603–14. Epub 2015/07/16. doi: 10.1074/jbc.M115.651604 ; PubMed Central PMCID: PMC4571884. 26175156 PMC4571884

[76] Kawai T, Elliott KJ, Scalia R, Eguchi S. Contribution of ADAM17 and related ADAMs in cardiovascular diseases. Cell Mol Life Sci. 2021;78(9):4161–87. Epub 2021/02/13. doi: 10.1007/s00018-021-03779-w . 33575814 PMC9301870

[77] Calligaris M, Cuffaro D, Bonelli S, Spano DP, Rossello A, Nuti E, et al. Strategies to Target ADAM17 in Disease: From its Discovery to the iRhom Revolution. Molecules. 2021;26(4). Epub 2021/02/14. doi: 10.3390/molecules26040944 ; PubMed Central PMCID: PMC7916773. 33579029 PMC7916773

[78] Palau V, Riera M, Soler MJ. ADAM17 inhibition may exert a protective effect on COVID-19. Nephrol Dial Transplant. 2020;35(6):1071–2. Epub 2020/04/16. doi: 10.1093/ndt/gfaa093 ; PubMed Central PMCID: PMC7184459. 32291449 PMC7184459

[79] Pearce SC, Coia HG, Karl JP, Pantoja-Feliciano IG, Zachos NC, Racicot K. Intestinal in vitro and ex vivo Models to Study Host-Microbiome Interactions and Acute Stressors. Front Physiol. 2018;9:1584. Epub 2018/11/30. doi: 10.3389/fphys.2018.01584 ; PubMed Central PMCID: PMC6240795. 30483150 PMC6240795

[80] Sekabira R, McGowan I, Yuhas K, Brand RM, Marzinke MA, Manabe YC, et al. Higher colorectal tissue HIV infectivity in cisgender women compared with MSM before and during oral preexposure prophylaxis. AIDS. 2021;35(10):1585–95. Epub 2021/04/09. doi: 10.1097/QAD.0000000000002907 ; PubMed Central PMCID: PMC8483241. 33831911 PMC8483241

[81] Mu WS M.; Haymans R.; Ritou E.; Rezek V.; Hamid P.; Kossyvakis A.; Sen Roy S.; Grijalva V.; Chattopadhyay A.; Meriwether D.; Kitchen S.G.; Fogelman A.M.; Reddy S.T.; Kelesidis T. ApoA-I mimetics reduce systemic and gut inflammation in chronic treated HIV. AIDS. 2020;[in press]. 10.1371/journal.ppat.1010160 PMC8740974 34995311

[82] Graham LS, Parhami F, Tintut Y, Kitchen CM, Demer LL, Effros RB. Oxidized lipids enhance RANKL production by T lymphocytes: implications for lipid-induced bone loss. ClinImmunol. 2009;133(2):265–75. 10.1016/j.clim.2009.07.011 PMC2805272 19699688

[83] Dionne S, Laberge S, Deslandres C, Seidman EG. Modulation of cytokine release from colonic explants by bacterial antigens in inflammatory bowel disease. Clin Exp Immunol. 2003;133(1):108–14. Epub 2003/06/26. doi: 10.1046/j.1365-2249.2003.02191.x ; PubMed Central PMCID: PMC1808749. 12823284 PMC1808749

[84] Trapecar M, Khan S, Roan NR, Chen TH, Telwatte S, Deswal M, et al. An Optimized and Validated Method for Isolation and Characterization of Lymphocytes from HIV+ Human Gut Biopsies. AIDS Res Hum Retroviruses. 2017;33(S1):S31–S9. Epub 2017/09/09. doi: 10.1089/AID.2017.0208 ; PubMed Central PMCID: PMC5684666. 28882052 PMC5684666

[85] Imaizumi S, Grijalva V, Navab M, Van Lenten BJ, Wagner AC, Anantharamiah GM, et al. L-4F Differentially Alters Plasma Levels of Oxidized Fatty Acids Resulting in More Anti-inflammatory HDL in Mice. Drug Metab Lett. 2010. doi: 10.2174/187231210791698438 20642447 PMC3037264

[86] Stevens JR, Al Masud A, Suyundikov A. A comparison of multiple testing adjustment methods with block-correlation positively-dependent tests. PLoS One. 2017;12(4):e0176124. Epub 2017/04/30. doi: 10.1371/journal.pone.0176124 ; PubMed Central PMCID: PMC5409054. 28453517 PMC5409054

