# Supplementary figures and images for: ApoA-I mimetics reduce systemic and gut inflammation in chronic treated HIV
Source: PLoS Pathog. 2022 Jan 7;18(1):e1010160. doi: 10.1371/journal.ppat.1010160 (PMC8740974; doi:10.1371/journal.ppat.1010160)

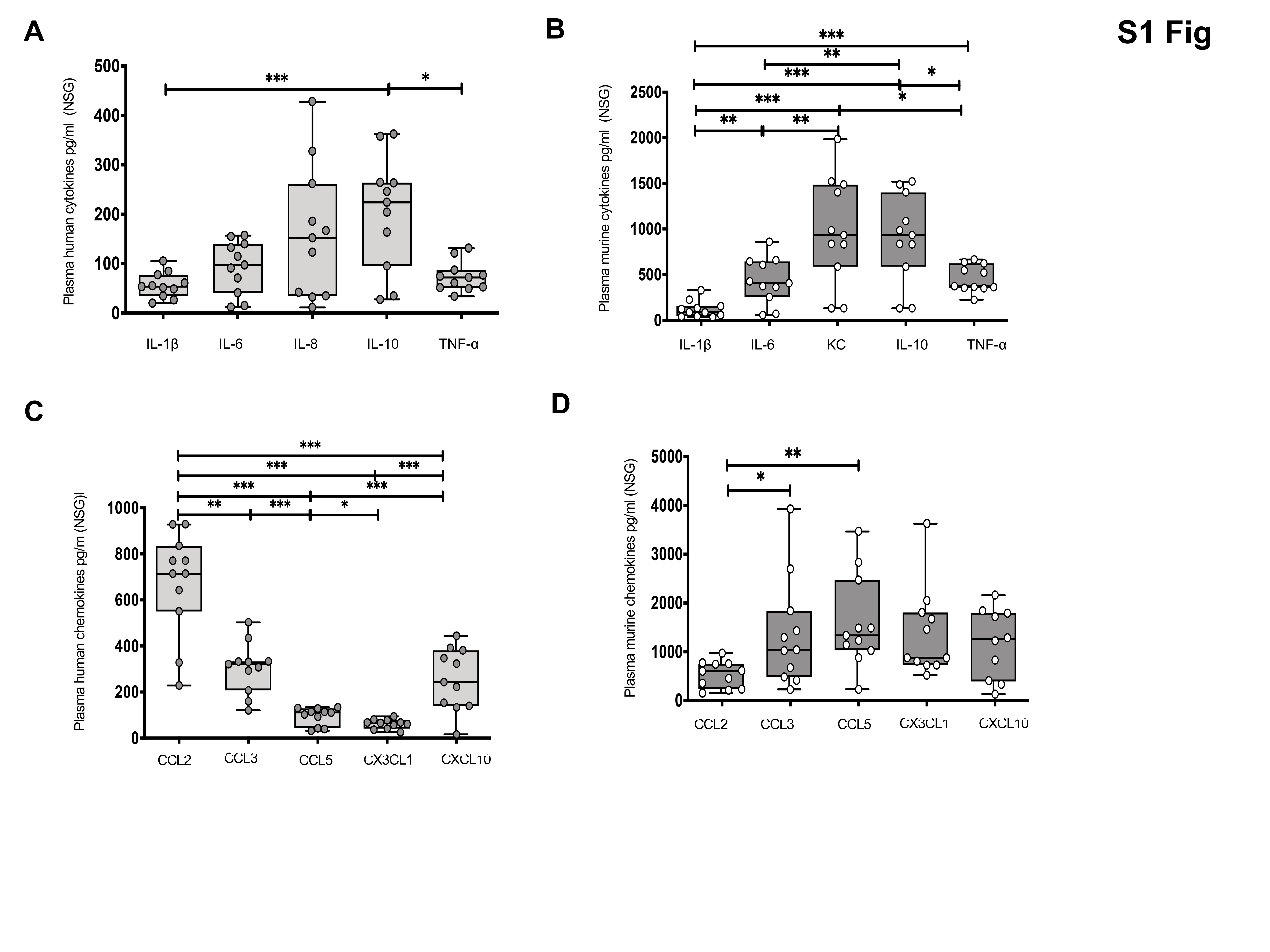

Supplement: S1 Fig — NSG humanized (BLT) mice were constructed and treated with control transgenic tomato concentrate (n = 11). Human and murine cytokines [interleukin (IL)-1β, IL-6, IL-8, IL-10, tumor necrosis factor alpha (TNF-α)] and chemokines [C-C Motif Chemokine Ligand 2 (CCL2), CCL3, CCL5, C-X3-C Motif Chemokine Ligand 1 (CX3CL1), C-X-C Motif Chemokine Ligand 10 (CXCL10)] were determined in plasma by Luminex immunoassays. A-D. Levels (pg/ml) of human (A) and murine (B) cytokines and human (C) and murine (D) chemokines in plasma from blood isolated from uninfected NSG (n = 11) BLT mice after 16 weeks of HIV infection in the HIV+ART + group. Data represent box and whiskers with minimum, median and maximum values (n = 11 mice per group). Datapoints represent mean of at least 2 experimental replicates per mouse. The Kruskal Wallis was used to compared >2 groups and the Mann-Whitney test was used to compare 2 groups (*p < 0.05, **p < 0.01, ***p < 0.001). (TIF) [file ppat.1010160.s002.tif]

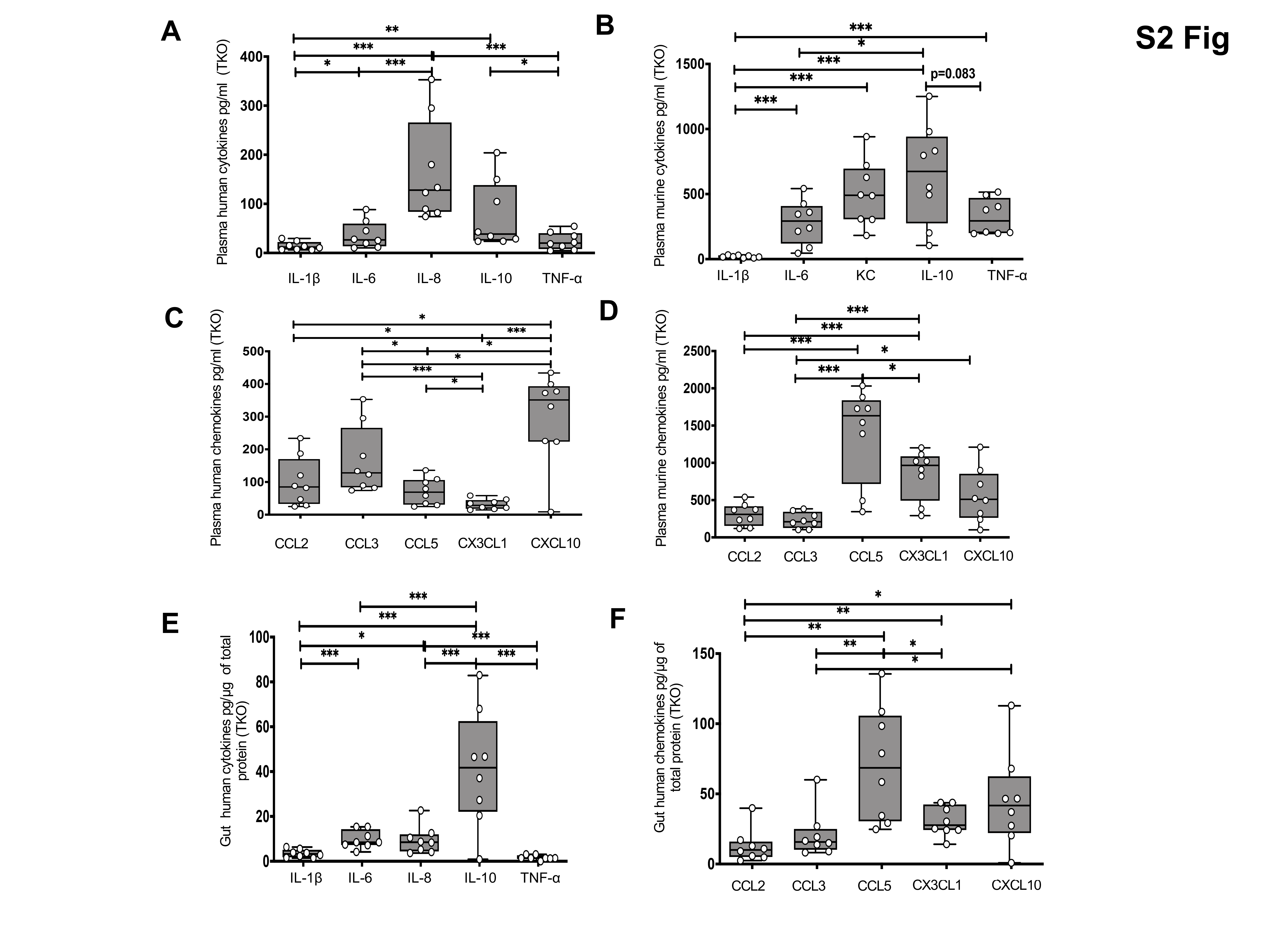

Supplement: S2 Fig — TKO C57 humanized (BLT) mice were constructed and treated with control transgenic tomato concentrate. Blood and small intestine from each mouse were collected and plasma and protein lysates were prepared from uninfected TKO (n = 8) BLT mice after 16 weeks of HIV infection in the HIV+ART + group. Human and murine cytokines [interleukin (IL)-1β, IL-6, IL-8, IL-10, tumor necrosis factor alpha (TNF-α)] and chemokines [C-C Motif Chemokine Ligand 2 (CCL2), CCL3, CCL5, C-X3-C Motif Chemokine Ligand 1 (CX3CL1), C-X-C Motif Chemokine Ligand 10 (CXCL10)] were determined in plasma and gut tissue lysates by Luminex immunoassays. A-D. Levels (pg/ml) of human (A) and murine (B) cytokines and human (C) and murine (D) chemokines in plasma from blood isolated from uninfected TKO BLT mice. E, F. Levels (pg/μg of total protein) of human cytokines (E) and chemokines (F) in tissue lysates from gut isolated from uninfected TKO (n = 8) BLT mice after 16 weeks of HIV infection in the HIV+ART + group. Data represent box and whiskers with minimum, median and maximum values (n = 8 mice per group). Datapoints represent mean of at least 2 experimental replicates per mouse. The Kruskal Wallis was used to compared >2 groups and the Mann-Whitney test was used to compare 2 groups (*p < 0.05, **p < 0.01, ***p < 0.001). (TIF) [file ppat.1010160.s003.tif]

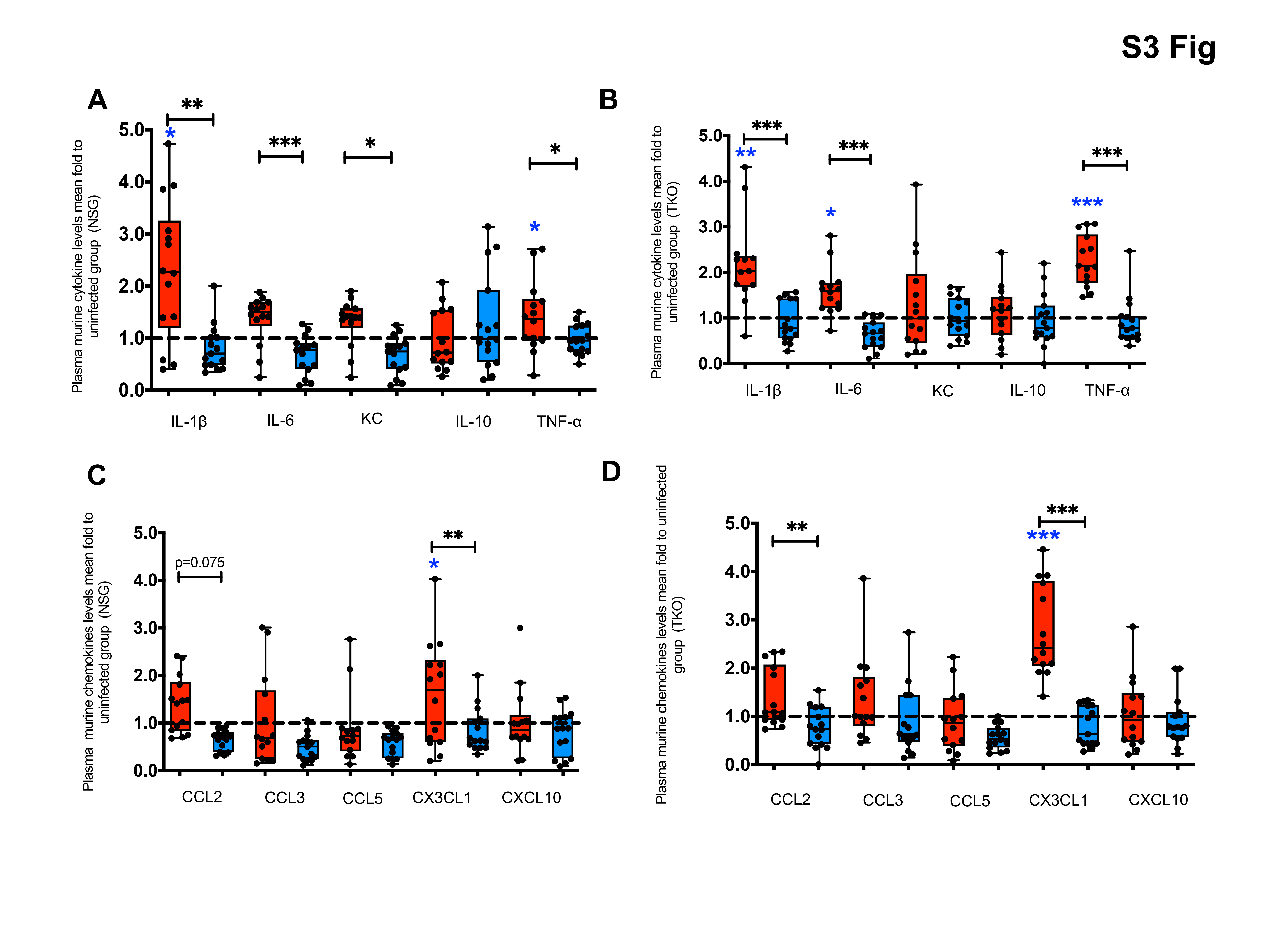

Supplement: S3 Fig — NSG (n = 51) and TKO (n = 37) humanized mice were constructed, infected with HIV and treated with antiretroviral therapy (ART), and control transgenic tomato concentrate and/or oral ApoA-I mimetic peptide 6F in the form of transgenic tomato (Tg6F). Murine cytokines [interleukin (IL)-1β, IL-6, KC/murine IL-8 homolog, IL-10, tumor necrosis factor alpha (TNF-α)] and chemokines [C-C Motif Chemokine Ligand 2 (CCL2), CCL3, CCL5, C-X3-C Motif Chemokine Ligand 1 (CX3CL1), C-X-C Motif Chemokine Ligand 10 (CXCL10)] were determined in plasma by Luminex immunoassays after 16 weeks of HIV infection in the HIV+ART + group. The mean value of each measurement in HIV+ART+ (red color) and HIV+ART+/Tg6F+ (blue color) mice BLT mice was expressed as fold to the mean value of each measurement in uninfected BLT mice (within the same cohort). A. Fold changes of murine cytokines in plasma of HIV+ART+ (n = 19) and HIV+ART+/Tg6F+ (n = 21) NSG mice compared to uninfected (n = 11) BLT mice. B. Fold changes of murine cytokines in plasma of HIV+ART+ (n = 14) and HIV+ART+/Tg6F+(n = 15) TKO mice compared to uninfected (n = 8) BLT mice. C. Fold changes of murine chemokines in plasma of HIV+ART+ (n = 19) and HIV+ART+/Tg6F+ (n = 21) NSG mice compared to uninfected (n = 11) BLT mice. D. Fold changes of murine chemokines in plasma of HIV+ART+ (n = 14) and HIV+ART+/Tg6F+(n = 15) TKO mice compared to uninfected (n = 8) BLT mice. Data represent box and whiskers with minimum, median and maximum values (n = 8–21 mice per group). Datapoints represent mean of at least 2 experimental replicates per mouse. The Kruskal Wallis was used to compared >2 groups and the Mann-Whitney test was used to compare 2 groups (*p < 0.05, **p < 0.01, ***p < 0.001). The asterisks in blue demonstrate the comparison relative to the uninfected group. A. Levels (pg/ml) of murine cytokines [interleukin (IL)-1β, IL-6, KC/murine IL8 homolog, IL-10, tumor necrosis factor alpha (TNF-α)] in plasma from uninfected NSG BLT mice after 16 weeks [file ppat.1010160.s004.tif]

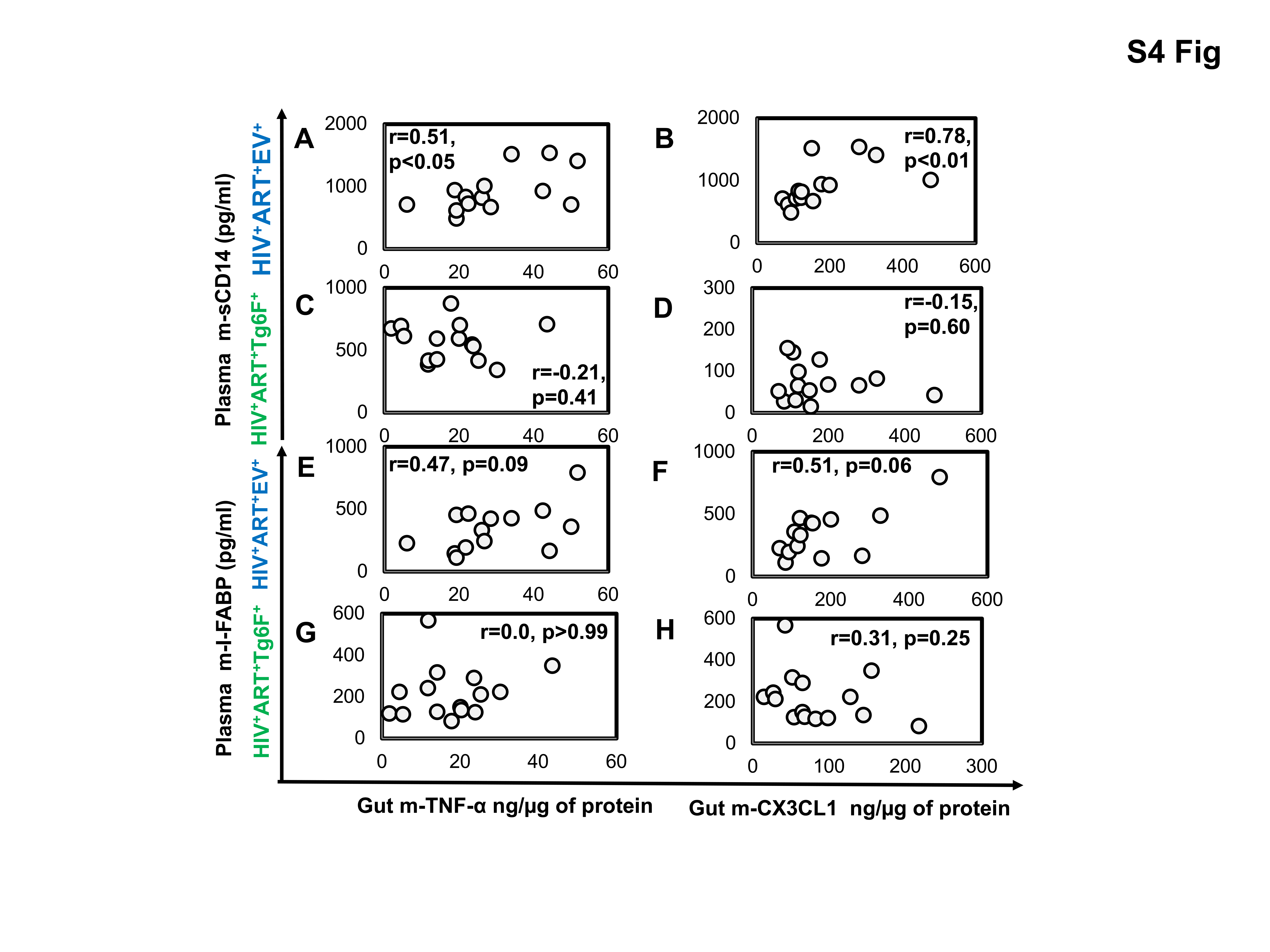

Supplement: S4 Fig — TKO C57 (n = 45) humanized mice were constructed, infected and treated with antiretroviral therapy (ART), and control transgenic tomato concentrate (EV) and/or oral ApoA-I mimetic peptide 6F in the form of transgenic tomato (Tg6F). Murine TNF-α and CX3CL1 were determined by Luminex immunoassays as in Materials and Methods. Plasma murine sCD14 and I-FABP are biomarkers of “leaky gut” that predict morbidity in chronic treated HIV and were determined by ELISAs. Scatter plots of gut protein levels of murine TNF-α and CX3CL1 (x axis) against biomarkers of “leaky gut” (y axis) are shown for the ART-treated groups and the ART+Tg6F groups (n = 8–22 mice per group). The Spearman correlation coefficient was used for all correlations between i) plasma sCD14 with protein levels of gut TNF-α, (A, C), gut m-CX3CL1 (B, D), in HIV+ART+ treated mice (A, B), and HIV+ART+Tg6F+ treated mice (C, D); ii) plasma I-FABP with protein levels of gut TNF-α (E, G), gut m-CX3CL1, (F, H), in HIV+ART+ treated mice, (E, F) and HIV+ART+Tg6F+ treated mice (G, H). (TIF) [file ppat.1010160.s005.tif]

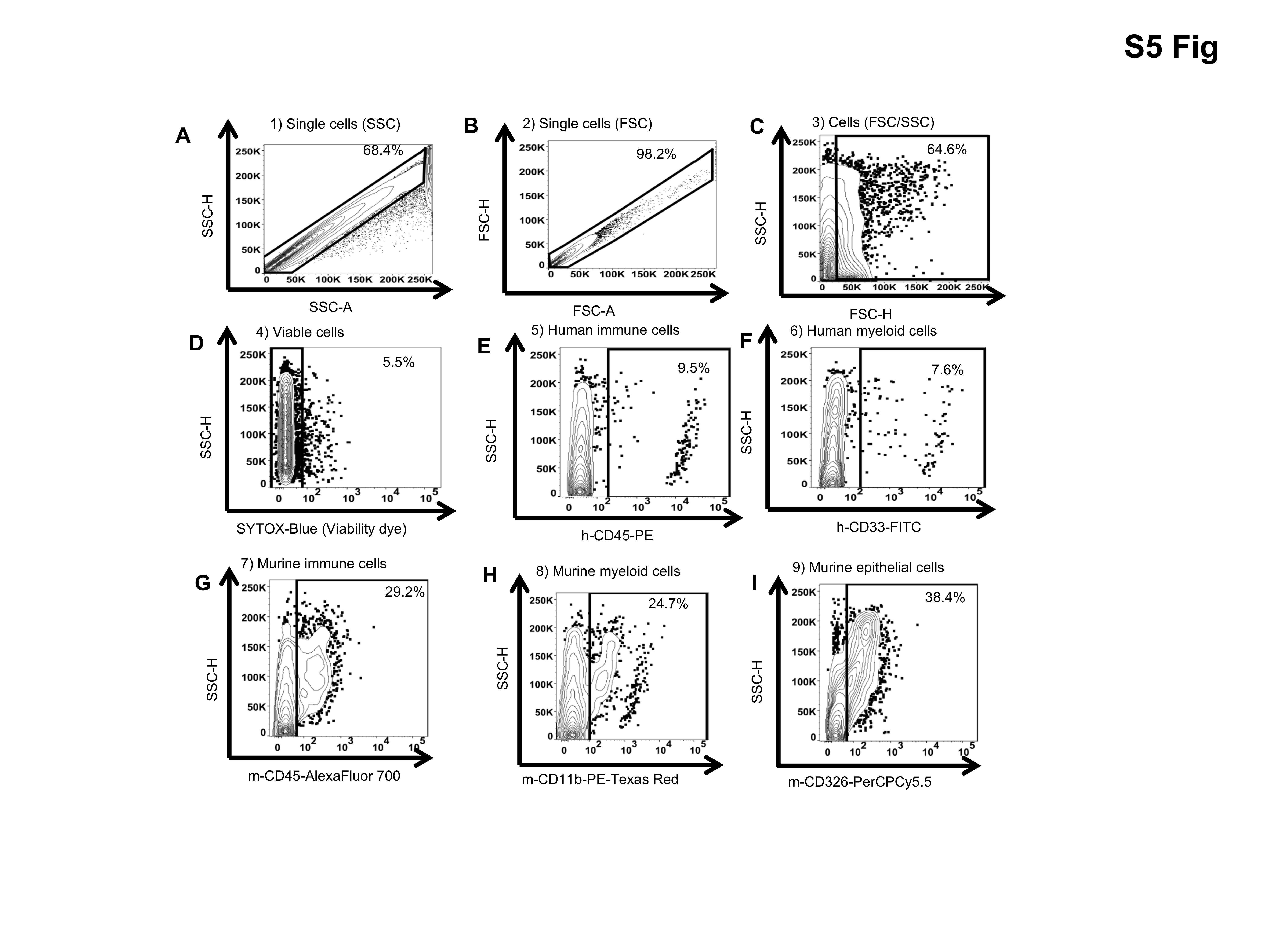

Supplement: S5 Fig — A-I. Gating strategy in flow cytometry experiments in intestinal cells of humanized mice. TKO C57 (n = 45) humanized (BLT) mice were constructed, infected with HIV and treated with antiretroviral therapy (ART) and control transgenic tomato concentrate and/or oral ApoA-I mimetic peptide 6F in the form of transgenic tomato (Tg6F). Single cell suspension from small intestine was prepared and was used for flow cytometry in intestinal cells. Fluorescence intensity of a positive cell population was compared to a negative cell population (fluorescence minus one negative control for staining) (ΔMFI). Representative flow cytometry data from gut single cell suspension of TKO BLT mice: A. 1) Single cells (SSC); B. ➔ 2) Single cells (FSC); C. 3) Cells (FSC/SSC) to exclude debris; D. ➔ 4) Viable cells were gated as negative stain for the SYTOX Blue dead cell stain; E. 5) Human immune cells were gated as hCD45+ on gate 4. F. 6) Human myeloid cells were gated as hCD33+ on hCD45+ cells on gate 5; G. 7) Murine immune cells were gated as mCD45+ on gate 4. H. 8) Murine myeloid cells were gated as hCD11b+ on mCD45+ cells on gate 7; I. 9) Murine epithelial cells were gated as m-CD326+ on mCD45- cells on gate 7. 10) Murine endothelial cells were gated as mCD31+ on m-CD326-, m-CD45- cells on gate 9 (not shown). (TIF) [file ppat.1010160.s006.tif]

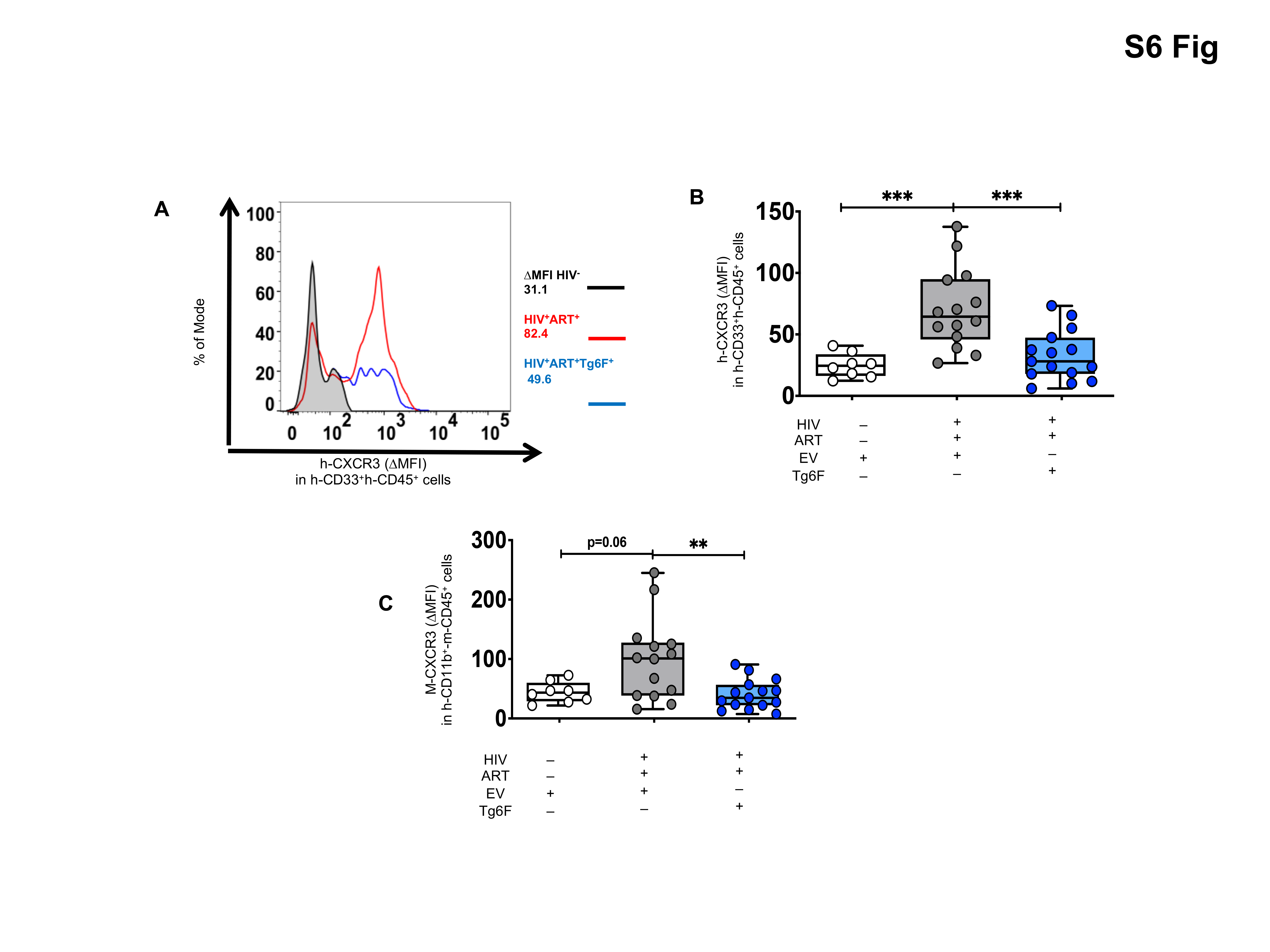

Supplement: S6 Fig — Single cell suspension from gut was prepared and cellular membrane protein levels of human CXCR3 (h-CXCR3) in human CD33+CD45+ myeloid immune cells were determined by flow cytometry. A. Representative data of median fluorescence intensity (MFI) of a positive cell population compared to a negative cell population (fluorescence minus one negative control for staining shown in light filled grey histogram)] of h-CXCR3 in human h-CD33+h-CD45+ myeloid immune cells are shown. B. Summary of data (ΔMFI h-CXCR3) for (A). C. Summary of data for ΔMFI m-CXCR3 in murine m-CD11b+CD45+ myeloid immune cells. Data represent box and whiskers with minimum, median and maximum values (n = 8–22 mice per group). The Mann-Whitney test was used to compare 2 groups (*p < 0.05, **p < 0.01, ***p < 0.001). (TIF) [file ppat.1010160.s007.tif]
