# Supplementary material for: ApoA-I mimetics reduce systemic and gut inflammation in chronic treated HIV
Source: PLoS Pathog. 2022 Jan 7;18(1):e1010160. doi: 10.1371/journal.ppat.1010160 (PMC8740974; doi:10.1371/journal.ppat.1010160)
Supplement: S1 Table — Spearman correlation coefficient (r) is shown for all associations between pairs. Statistically significant associations (p<0.05) are indicated in bold. Trends for associations (0.05<p<0.10) are underlined in italics. (PDF) [file ppat.1010160.s008.pdf]

Supporting information

Supplemental Table 1

S1 Table: Associations of protein levels (% of positive cells and ΔMFI) of ADAM-17 in human CD33+ myeloid cells and murine CD326+ epithelial cells with gut oxidized lipoproteins (HDLox, LDLox), plasma m-IFABP, human and murine sCD14 and sCD163 levels in HIV+/ART treated TKO BLT mice (n=14) and HIV+/ART/Tg6F (n=15) treated TKO BLT mice. Spearman correlation coefficient (r) is shown for all associations between pairs. Statistically significant associations ($p<0.05$) are indicated in bold. Trends for associations ($0.05<p<0.10$) are underlined in italics.

S1 Table

	ΔMFI m- ADAM17 in $CD326^+$ cells			
	HIV^+ART^+		$HIV^+ART^+Tg6F^+$	
	**r**	p	**r**	p
**Gut HDLox (pg/ml)**	**0.54**	**0.030**	0.13	0.202
**Gut LDLox (pg/ml)**	0.39	0.121	0.06	0.404
**m-IFABP pg/ml**	**0.49**	**0.032**	0.12	0.134
**m-sCD14 pg/ml**	**0.55**	**0.034**	0.16	0.202
**m-sCD163 pg/ml**	0.44	0.058	0.04	0.323
**h-sCD14 pg/ml**	**0.57**	**0.014**	0.13	0.172
**h-sCD163 pg/ml**	0.37	0.103	0.04	0.742
